# Elevated retrotransposon activity and genomic instability in primed pluripotent stem cells

**DOI:** 10.1186/s13059-021-02417-9

**Published:** 2021-07-09

**Authors:** Haifeng Fu, Weiyu Zhang, Niannian Li, Jiao Yang, Xiaoying Ye, Chenglei Tian, Xinyi Lu, Lin Liu

**Affiliations:** 1 State Key Laboratory of Medicinal Chemical Biology, Nankai University, Tianjin, China; 2 Department of Cell Biology and Genetics, College of Life Sciences, Nankai University, Tianjin, China; 3 The Key Laboratory of Bioactive Materials Ministry of Education, Nankai University, Tianjin, China; 4 College of Pharmacy, Nankai University, 94 Weijin Road, Tianjin, 300071 China

**Keywords:** Naïve and primed pluripotent state, Telomeres, 2C genes, Genome stability, Retrotransposons, Histone modifications

## Abstract

**Background:**

Naïve and primed pluripotent stem cells (PSCs) represent two different pluripotent states. Primed PSCs following in vitro culture exhibit lower developmental potency as evidenced by failure in germline chimera assays, unlike mouse naïve PSCs. However, the molecular mechanisms underlying the lower developmental competency of primed PSCs remain elusive.

**Results:**

We examine the regulation of telomere maintenance, retrotransposon activity, and genomic stability of primed PSCs and compare them with naïve PSCs. Surprisingly, primed PSCs only minimally maintain telomeres and show fragile telomeres, associated with declined DNA recombination and repair activity, in contrast to naïve PSCs that robustly elongate telomeres. Also, we identify LINE1 family integrant *L1Md_T* as naïve-specific retrotransposon and ERVK family integrant *IAPEz* to define primed PSCs, and their transcription is differentially regulated by heterochromatic histones and Dnmt3b. Notably, genomic instability of primed PSCs is increased, in association with aberrant retrotransposon activity.

**Conclusions:**

Our data suggest that fragile telomere, retrotransposon-associated genomic instability, and declined DNA recombination repair, together with reduced function of cell cycle and mitochondria, increased apoptosis, and differentiation properties may link to compromised developmental potency of primed PSCs, noticeably distinguishable from naïve PSCs.

**Supplementary Information:**

The online version contains supplementary material available at 10.1186/s13059-021-02417-9.

## Background

Murine embryonic stem cells (mESCs) from preimplantation embryos resemble an earlier stage in development known as naïve pluripotent state. Primed epiblast stem cells (mEpiSCs) are derived from murine post-implantation epiblast embryos commonly known to be in primed pluripotent state [1, 2]. Human ESCs derived from preimplantation embryos are transcriptionally similar to mEpiSCs [2–4]. Naïve mESCs can be sustained in the presence of serum and leukemia inhibitory factor (LIF) condition [5], while the mEpiSCs can be maintained in the presence of FGF2 and Activin A condition [1, 2]. Naïve mouse pluripotent stem cells (mPSCs) exhibit germline competence as determined by chimera production test and can generate all-PSC mice by tetraploid embryo complementation (TEC) test, the most stringent functional assay of naïve pluripotency [6–9]. In contrast, primed mEpiSCs fail in germline competence and even rarely produce chimeras [1, 2], suggesting their reduced developmental and differentiation capacity. Under defined in vitro culture conditions, the primed mouse epiblast-like cells (mEpiLCs) can be established from naïve mESCs [10–12]. Naïve and primed pluripotent states exhibit different molecular signatures in the epigenome and transcriptome profile [3, 10, 11, 13–18].

Moreover, a “ground state” beyond naïve state has been achieved by culture under 2i/LIF condition (2i, GSK3, and MEK1/2 inhibitors) [19]. 2i reduces the DNA methyltransferases Dnmt3a/3b to promote hypomethylation and enhances Tet1/2 activity (consequently, increased 5-hydroxymethylcytosine (5hmC) levels) and “passive” loss of DNA methylation, resulting in a distinct transcriptional and epigenetic state that includes uniform expression of key pluripotency factors, such as Nanog and Prdm14 [20, 21]. Ground-state cells exhibit lower expression of lineage affiliated genes, reduced prevalence at promoters of the repressive histone modification H3K27me3, and fewer bivalent domains, which are thought to mark genes poised for either up- or downregulation [22]. Furthermore, female PSCs under the ground state or naïve state show both X chromosome activation, but inactivate one of the X chromosomes under primed state [18]. mESCs under 2i culture conditions also can produce all-ESC pups but prolonged inhibition with 2i could damage the developmental potential [23–25].

Notably, transposable elements, particularly endogenous retroviruses (ERVs) that have important roles in pluripotency, can wire the pluripotency network in humans and mice [26–33]. Naïve mouse ESCs periodically activate endogenous retrovirus (ERVs) and 2 cell (2C) genes [28]. ERVs together with other retrotransposons, including long interspersed nuclear elements (LINEs) and short interspersed nuclear elements (SINEs) occupy approximately 40% of mammalian genomes, and mostly are conserved and evolved [34–36]. Nevertheless, retrotransposition of active LINEs and ERVs could induce genomic instability, disrupt regulatory elements, cause mutations, or drive genome evolution [34, 37]. Therefore, the repression of retrotransposons is critical for the maintenance of genome stability in ESCs [38]. For instance, Kap1 and histone 3.3 repress ERVs in mESCs [38–40]. In addition, telomere elongation and maintenance are essential for unlimited self-renewal and pluripotency of PSCs [41, 42]. Short telomeres impair differentiation capacity of ESCs [43]. Yet, regulation of transposable elements and telomere length in primed PSCs remains largely unclear.

In this study, we sought to delineate telomere and retrotransposon features association with the genomic stability of primed PSCs in comparison with those of naïve PSCs in mice, given that the two states have been functionally defined by developmental pluripotency in vivo.

## Results

### Conversion of naïve mESCs to primed mEpiSCs results in repression of DNA recombination repair pathway

Naïve mESCs with distal Oct4 GFP fluorescence were maintained in serum/LIF medium on mitomycin C-inactivated MEFs served as feeder cells. The GFP reporter fluorescence driven by distal *Oct4* promoter and enhancer can indicate the naïve state of pluripotency [4, 44–46]. We converted naïve mESCs into the primed state by culture in F/A medium (Fgf2 + ActivinA) based on the method described previously [1, 11, 18, 47]. Primed mEpiSCs also were cultured on feeder cells, like naïve mESCs [25]. Domed shaped naïve ES colonies began to flatten within 48 h following culture in F/A medium, as shown in a previous report [11].

**Fig. 1:**
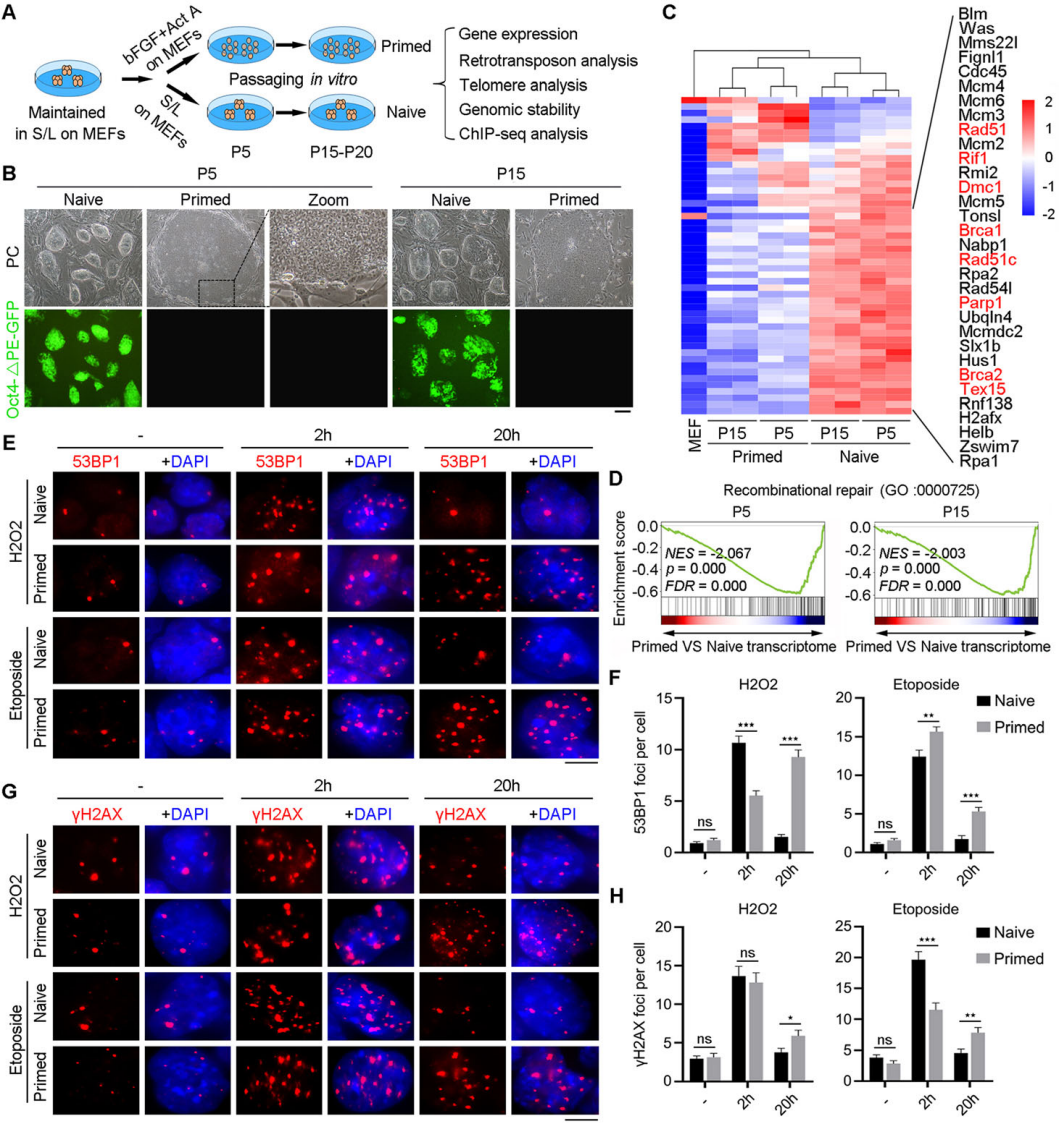
Conversion from naïve to primed state suppresses DNA recombination repair pathway. **A** Schematic diagram illustrating conversion of naïve to primed PSCs and experimental design. **B** Representative morphology of naïve mESCs and primed mEpiSCs at passage P5 and P15 under bright field with phase contrast (PC) optics and expression of Oct4-△PE-GFP fluorescence. Scale bar = 100 μm. **C** Heatmap highlighting gene expression profile related to the DNA recombination and repair pathway in naïve and primed cells at P5 and P15. The representative genes are listed on the right. **D** Gene set enrichment analysis (GSEA) indicating that downregulated genes under primed state are highly enriched in the DNA recombination and repair pathway. **E, F** DNA damage responses by exposure to 2.5 μM etoposide and 5 mM H_2_O_2_ for 2 h followed by immunofluorescence of 53BP1 foci at 2 and 20 h recovery. Scale bar = 5 μm. **F** The quantitative results of **E**. More than 50 cells were randomly counted. **G, H** DNA damage responses by exposure to 2.5 μM etoposide and 5 mM H_2_O_2_ for 2 h followed by immunofluorescence of γH2AX foci at 2 and 20 h recovery. Scale bar = 5 μm. **H** The quantitative results of **G**. More than 50 cells were randomly counted. Data is shown as Mean ± SEM. *P < 0.05; **P < 0.01; ***P < 0.001

To achieve stable state, we expanded the cells for five passages (P5, relatively early passages) and also for additional 10 or 15 passages (P15 or P20, relatively late passages) under naïve and primed conditions (Fig. 1a). Primed mEpiSCs grew as flat but larger colonies and were thus morphologically distinctive from naïve mESCs showing compact, dome-shaped but smaller colonies (Fig. 1b; and Additional file 1: Figure S1a). The GFP reporter fluorescence indicating the naïve state of pluripotency extinguished following conversion into primed state (Fig. 1b). Analysis of cell cycle at P5 showed that cells under primed state displayed extended G1 phase (Additional file 1: Figure S1b), in contrast to naïve mESCs with shorter G1 phase [48, 49]. The primed cells expressed two core pluripotent factors Oct4 and Nanog, but at levels lower than those of naïve cells (Additional file 1: Figure S1c, e, f), as shown previously [11, 12, 18]. Additionally, expression of an important naïve marker SSEA1 was reduced in primed cells (Additional file 1: Figure S1e, f), whereas *Dnmt3b*, a marker gene of primed state [2], was upregulated in primed cells (Additional file 1: Figure S1d, g). By RNA-seq analysis, naïve mESCs highly expressed known naïve marker genes such as *Rex1*, *Stella*, *Esrrb*, *Tbx3*, and *Nr0b1* [3], while marker genes for primed state such as *Otx2*, *Oct6*, *Fgf5*, *T*, *Cer1*, and *Pitx2* [2] were upregulated at either passage 5 or 15 (Additional file 1: Figure S1h). Our RNA-seq analysis also revealed differential global transcription profiles of the two pluripotent states (Additional file 1: Figure S1i; Additional file 2: Table S1; Additional file 3: Table S2, Padj < 0.01, fold change ≥ 2).

To validate the developmental potential of mouse naïve and primed cells, we performed chimera formation and tetraploid embryo complementation (TEC) assays. Primed mEpiSCs rarely can form chimeras (Additional file 1: Figure S2a, b, c), while naïve mESCs generated germline chimera mice and completely ESC pups by TEC assay (Additional file 1: Figure S2d, e, f) that were fertile (Additional file 1: Figure S2g). These results further support the notion that the developmental pluripotency of naïve PSCs is high, distinct from primed PSCs.

Naïve and primed PSCs exhibit different gene expression profiles and signaling pathways [3, 18]. By Kyoto Encyclopedia of Genes and Genomes (KEGG) analysis, the most upregulated genes in naïve cells at P5 and P15 were enriched in signaling pathways regulating pluripotent stem cells (Additional file 1: Figure S3a, b), demonstrating that molecular pluripotency of naïve cells is indeed higher than that of primed cells. Signaling pathways that affect pluripotency such as MAPK, Wnt, and PI3K-Akt, and cell adhesion molecules (CAMs) and cytokine-cytokine receptor interaction also differ between the two states (Additional file 1: Figure S3c, d). Moreover, the transcription programs of naïve and primed cells resemble their in vivo counterparts ICM and post-implantation epiblasts respectively in terms of cell cycle and apoptosis pathway (Additional file 1: Figure S3e, f).

Remarkably, by gene ontology (GO) analysis, DNA recombinational repair pathway was significantly downregulated under primed state (Fig. 1c, d). Expression levels of *Rif1*, *Rad51*, *Dmc1*, *Brca1*, and *Brca2* were remarkably decreased in primed cells (Fig. 1c), and these genes are involved in double-strand break (DSB) repair process or telomere maintenance [50–52]. Mismatch repair and nucleotide-excision repair pathways were also repressed in primed, compared with naïve mESCs, but non-homologous end joining (NHEJ) and base-excision repair pathways did not differ between the two states (Additional file 1: Figure S4a-h). These data imply the more robust DNA repair capacity likely suggestive of better genomic stability maintenance in naïve mESCs compared with those of primed mEpiSCs.

To assess whether primed cells are more susceptible to DNA damage response, we performed immunofluorescence microscopy of γH2AX and 53BP1, commonly used as DNA damage response markers [50, 53] in naïve and primed state PSCs by exposure to low dose of either etoposide or H_2_O_2_. We intuitively monitored 53BP1 and γH2AX foci by immunofluorescence in etoposide or H_2_O_2_-treated cells. Increased 53BP1 and γH2AX foci appeared at 2 h in both cell types, and the number of foci was significantly reduced likely indicative of DNA repair after 20 h recovery in naïve cells, and almost similar to that without exposure to the DNA damage at 0 h (Fig. 1e–h; Additional file 1: Figure S1j). However, more 53BP1 and γH2AX foci persisted in primed cells than in naïve cells at 20 h (Fig. 1e–h; Additional file 1: Figure S1j). These data conclude that the DNA repair capacity is robust in naïve cells and is reduced in primed state.

### Two-cell (2C) embryo genes are repressed in primed mEpiSCs

**Fig. 2:**
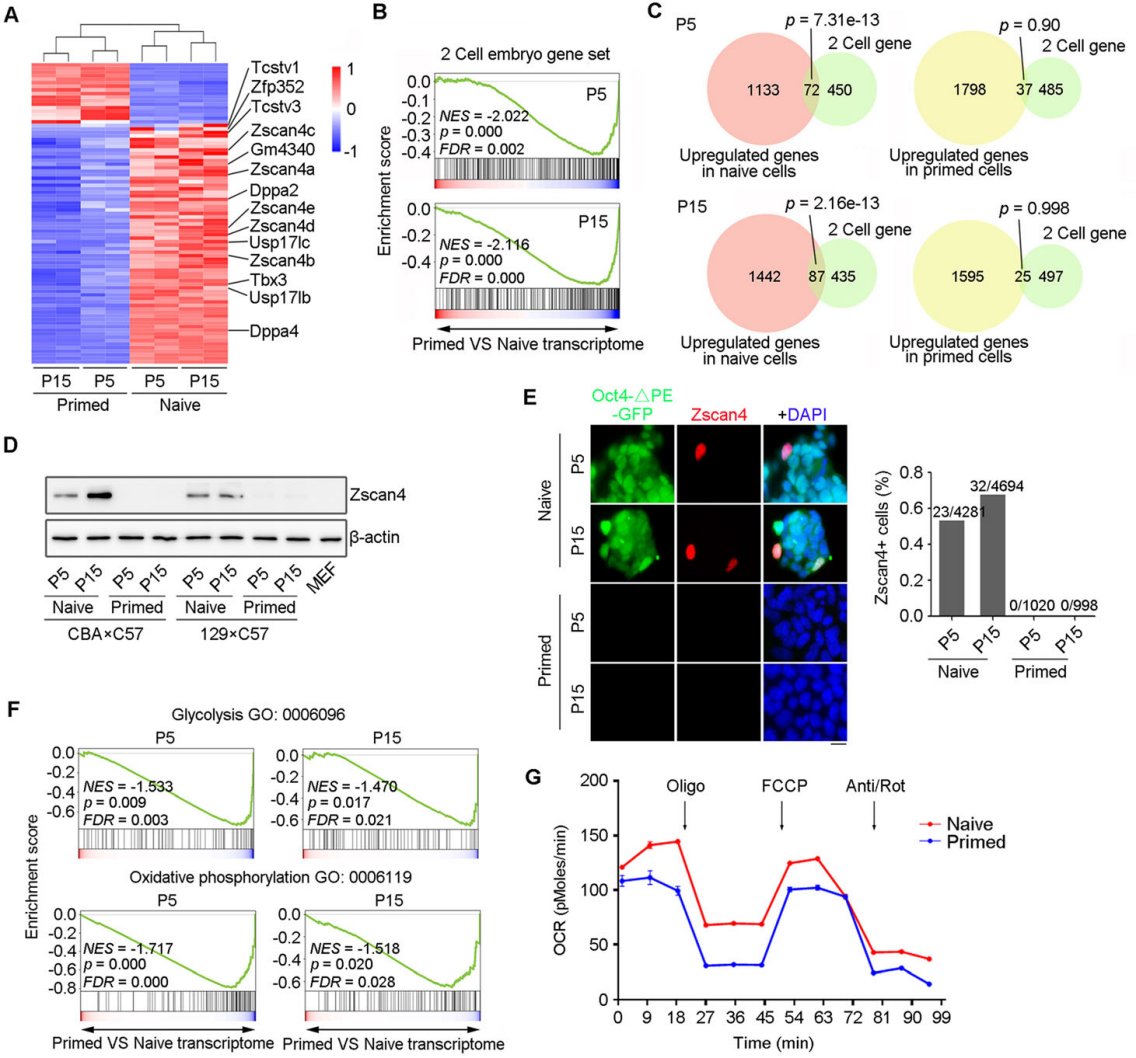
2C genes including *Zscan4* are repressed in primed mEpiSCs. **A** Heatmap highlighting gene expression profile related to 2C genes in CBA × C57 P5 and P15 naïve and primed cells. The representative genes are listed on the right. **B** Gene set enrichment analysis (GSEA) indicating that downregulated genes under primed state are highly enriched in the 2C gene set. **C** Venn diagram showing the overlap between upregulated genes in naïve and primed cells and 2C genes. *P* value was calculated by Fisher’s exact test. **D** Protein level of Zscan4 by western blot analysis of two different naïve and primed cell lines at P5 and P15. MEF cells served as a negative control. **E** Representative images of immunofluorescence of Zscan4 protein in CBA × C57 mESCs and mEpiSCs cultures at P5 and P15. Scale bar = 10 μm. Right panel, proportion of Zscan4^+^ cells based on immunofluorescence images. Number of cells counted is shown on the top of the bar. **F** Gene set enrichment analysis (GSEA) indicating that downregulated genes under primed state are highly enriched in the metabolic glycolysis pathway as well as in the metabolic oxidative phosphorylation pathway. **G** Oxygen consumption rate (OCR) in response to oligomycin, FCCP, and rotenone/antimycin of naïve and primed cells (n = 3)

Furthermore, representative 2C genes, e.g., *Zscan4*, *Tcstv1/3*, *Usp17l*, *Dppa2*, and *Dppa4*, that were highly upregulated in naïve mESCs, were noticeably downregulated in primed mEpiSCs (Fig. 2a). We took advantage of previously reported lists of upregulated genes in 2C-like ESCs [28], and compared expression levels of 2C genes in naïve and primed cells. Conversion to primed state led to decreased enrichment of 2C embryo gene sets regardless of passages (Fig. 2b). 2C-like genes significantly overlapped with the upregulated genes in naïve cells compared with primed cells (*p* = 7.31e−13 for P5 and *p* = 2.16e−13 for P15), but showed no correlation with the upregulated genes in primed cells (*p* = 0.90 for P5 and *p* = 0.998 for P15) (Fig. 2c). These data suggested that 2C genes were repressed in primed mEpiSCs. Western blot and immunofluorescence microscopy confirmed sporadic expression of Zscan4 protein in naïve but not in primed cells (Fig. 2d, e). These results were also repeated in another independent cell line at 129 × C57 genetic background (Additional file 1: Figure S5a, b). In addition, RNA-seq data and seahorse experiment further confirmed that the metabolic pathways of glycolysis and oxidative phosphorylation were decreased during the transition from naïve to primed state (Fig. 2f, g). Therefore, unlike naïve mESCs, primed mEpiSCs lack a 2C-like cell subpopulation.

### Primed PSCs do not elongate telomeres

**Fig. 3:**
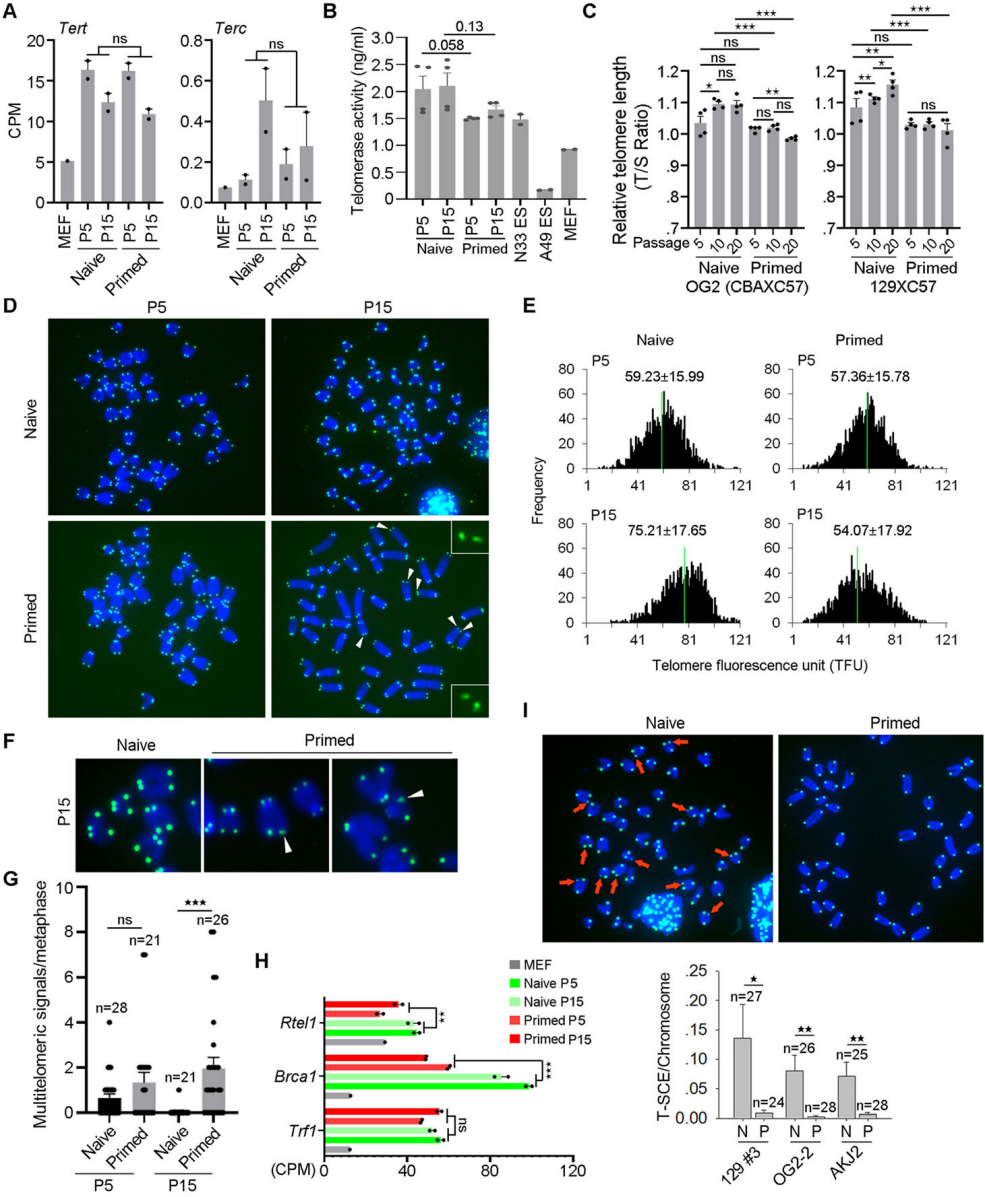
Distinct telomere maintenance in naïve and primed cells. **A** Expression levels (CPMs) of telomerase genes *Tert* and *Terc* in CBA × C57 naïve and primed cells at P5 and P15. Two biological repeats. **B** Quantification of telomerase activity of two different naïve and primed cell lines P5 and P15 by ELISA assay. WT N33 ESCs served as telomerase positive controls and telomerase-deficient G4 A49 ESCs served as negative controls. **C** Relative telomere length shown as T/S ratio by qPCR during passages (P5, P10, and P20) in naïve and primed cells in CBA × C57 or in 129 × C57 background. Four replicates are shown. *P < 0.05; **P < 0.01, ***P < 0.001. **D** Representative images displaying telomere FISH of naïve and primed cells at CBA × C57 background at P5 and P15. Blue, chromosomes stained with DAPI; green dots, telomeres. The white arrows indicate fragile telomeres. **E** Histogram shows distribution of relative telomere length displayed as TFU by Q-FISH analysis. Green line indicates medium telomere length. Mean ± s.d. of telomere length is shown above each panel. About 20 chromosome spreads were quantified for each group. Two independent experiments. **F** Enlarged telomere Q-FISH images, showing telomere fragility (white arrowhead) in primed cells but not in naïve cells. Telomeres were labeled with telomere PNA probes (green), and chromosomes labeled with DAPI (blue). **G** Frequency of telomere fragility per chromosome. n, number of spreads counted. Two independent experiments. **H** Expression levels (CPMs) of *Trf1*, *Brca1*, and *Rtel1* genes in MEF cells, naïve, and primed cells. Two biological repeats. **I** Representative micrographs showing telomere sister chromatid exchange (T-SCE, red arrows) in naïve and primed cells in CBA × C57 background by CO-FISH analysis. Bottom panel, frequency of T-SCE in the naïve and primed cell lines from three different genetic background. Two independent experiments. Data is shown as Mean ± SEM. *P < 0.05; **P < 0.01; ***P < 0.001

Primed PSCs do not express 2C genes and notably *Zscan4*, in contrast to naïve PSCs. *Zscan4* has been shown to be critical for telomere lengthening and maintaining the genomic stability of mESCs by telomere sister chromatid exchange (T-SCE)-based homologous recombination [52]. This prompted us to explore regulation of telomere maintenance of primed cells, compared with naïve cells. Telomerase genes *Tert* and *Terc* were expressed at similarly higher levels in naïve and primed cells, relative to MEF cells (Fig. 3a). In consistency, both pluripotent state cells expressed similarly high telomerase activity (Fig. 3b; Additional file 1: Figure S5c). Dynamics of relative telomere length revealed by qPCR, shown as telomere/single-copy gene (T/S) ratio demonstrated that telomeres lengthened in naïve mESCs but not in primed PSCs with increasing passages (Fig. 3c). By telomere Q-FISH, telomeres also lengthened significantly in naïve mESCs with passages, whereas primed mEpiSCs failed to elongate their telomeres but exhibited slightly shortened telomeres (Fig. 3d, e). Similar results were obtained in another cell line from 129 × C57 genetic background (Additional file 1: Figure S5d, e, f).

Notably, following relatively longer-term culture, primed cell lines at a later passage exhibited fragile telomeres (Fig. 3f, g; Additional file 1: Figure S5g). TRF1, RTEL1, and BRCA1 were reported to be involved in the formation of fragile sites [54–56]. *Trf1* expression level did not change between the two states, but *Rtel1* and *Brca1* expression levels were decreased in the primed cells compared to naïve cells (Fig. 3h). This, along with the declined DNA recombination repair capacity, might be linked to the fragile telomeres in primed mEpiSCs after longer passage. The fragile telomeres and declined recombination pathways are associated with minimal telomere maintenance in primed cells. These data indicated that vigorous telomere elongation takes place in naïve cells but not in primed cells.

Telomerase extends telomeres at a slow pace of about 50–100 nucleotides per cell cycle [57]. Although telomerase activity was high in both states, the difference in telomere length dynamics suggests that mechanism other than telomerase activity could be involved in telomere maintenance of these cells. Alternative lengthening of telomeres (ALT) is a telomerase-independent mechanism to elongate telomeres rapidly [58], in association with frequent telomere sister chromatic exchange (T-SCE), which can indicate the recombinational rate of telomeres [59–61]. We assessed T-SCE in naïve and primed cells by telomere chromosome orientation FISH (CO-FISH) analysis [59]. Frequency of T-SCE was extremely low in primed cells (Fig. 3i; Additional file 1: Figure S5h), associated with the declined expression level of *Dmc1* which is involved in telomere recombination process [52] (Fig. 1c).

To further explore whether the high expression of DNA recombination repair genes links to telomere maintenance, we knocked out *Brca1*, *Dmc1*, and *Rad51* in naïve cells respectively by CRISPR-Cas9 and measured telomeres (Additional file 1: Figure S6a, b). Cell morphology did not change much after knocking out each of these genes (Additional file 1: Figure S6c). Expression of pluripotent gene *Oct4* and 2C gene *Zscan4* also was not affected following knockout (KO) (Additional file 1: Figure S6d). Telomeres significantly shortened after *Dmc1* KO and slightly shortened after *Rad51* KO (Additional file 1: Figure S6e, f). Although telomere length slightly increased after *Brca1* KO, telomere fragility increased (Additional file 1: Figure S6e-h). Hence, higher expression levels of DNA recombination repair genes in naïve state facilitate telomere lengthening of mESCs, whereas low expression levels of DNA recombination repair genes in primed state may reduce telomere elongation.

DNA demethylation also could directly affect recombination level at telomere region, and increased DNA methylation level may inhibit telomere elongation [62, 63]. Expression levels of DNMTs varied in naïve and primed cells. *Dnmt3l* and *Dnmt3b*, as the naïve and primed marker genes [64, 65], were highly expressed in naïve and primed cells, respectively (Additional file 1: Figure S 7a). *Dnmt1*, *Uhrf1*, and *Dnmt3a* all were downregulated in primed cells (Additional file 1: Figure S7a). Expression levels of *Tet1* and *Tet2* were decreased, but *Tet3* increased in primed cells compared with naïve cells (Additional file 1: Figure S7a). Tet family proteins oxidize 5-methylcytosine (5mC) to 5-hydroxymethylcytosine (5hmC), an intermediate that can lead to DNA demethylation [66, 67]. By immunofluorescence microscopy and dot blot assay, 5mC level did not change, but 5hmC level was decreased in primed cells (Additional file 1: Figure S7b-d). Differences in DNA methylation/demethylation levels may also regulate telomeres of naïve and primed cells. Increased telomere recombination activity corroborates robust telomere lengthening in naïve PSCs.

### Retrotransposon transcription distinguishes primed from naïve PSCs

**Fig. 4:**
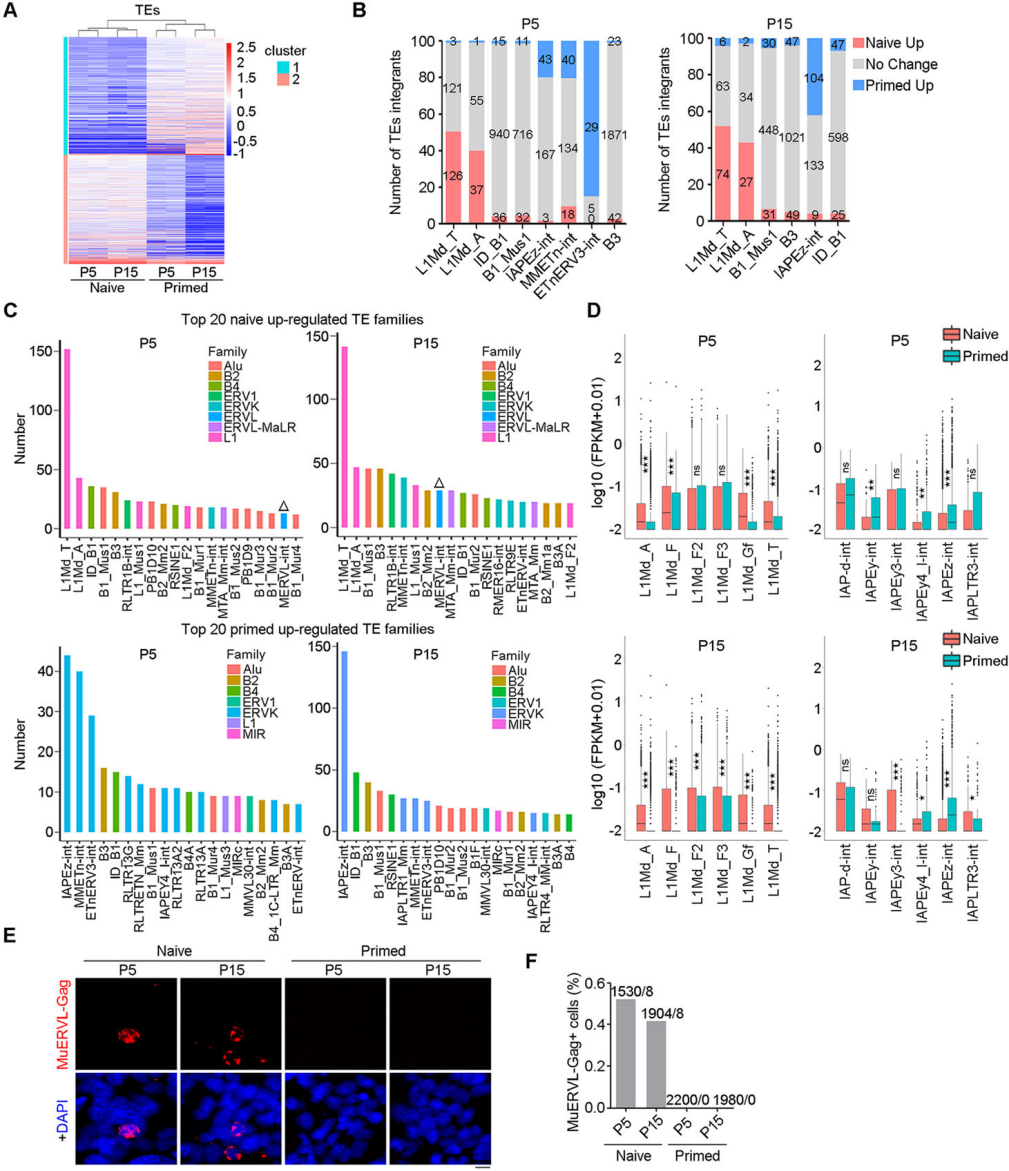
Retrotransposons distinguish mouse naïve from primed state. **A** Heatmap of RNA-seq expression data from CBA × C57 naïve mESCs and primed mEpiSCs. Data shown include 1000 differentiated expressed transposable elements (TEs) with the highest standard deviation (SD) between the samples. **B** TE signatures of naïve or primed mESCs. The most four heavily primed or naïve-biased TE families are represented as columns split into three segments: overexpressed integrants in naïve cells (1.5-fold cut-off, P < 0.01), overexpressed integrants in primed cells, and no change. **C** The top 20 TE families with the highest fold change between naïve and primed cells are heavily enriched for *LINE1* family (naïve) and *ERVK* family (primed). The triangles highlight the high expression of *MERVL* in naïve mESCs. **D** Left panel of boxplot showing the expression of integrant of *L1Md_A* and related families according to Dfam database. Right panel of boxplot showing the expression integrant of *IAPEz-int* and related families according to Dfam database. The significance is calculated by Wilcoxon Test. *P < 0.05; **P < 0.01; ***P < 0.001. **E** Representative immunofluorescence of 2C marker *MuERVL* Gag (red) in naïve and primed cells. DAPI-stained nuclei in blue. Scale bars = 10 μm. **F** Proportion of *MuERVL* Gag+ cells based on immunofluorescence images in Fig. 4E

Furthermore, we compared the transcription of transposable elements (TEs) in naïve and primed cells using an improved RNA-sequencing analysis pipeline [68]. The top 1000 TEs with the largest standard deviation (SD) separated naïve from primed cells (Fig. 4a). We looked at which TEs were overexpressed in naïve and primed cells, respectively. Top 20 TE families that were highly expressed in naïve cells at P5 and P15 included multiple types of retrotransposons but no DNA repeats (Fig. 4c). Among them, *L1* family members, especially *L1Md_Ts* were prominently upregulated in naïve cells compared with primed cells (Fig. 4b–d). Other *L1* family members, such as *L1Md_A*, *L1_Mus1*, and *L1Md_F2* were also upregulated in naïve mESCs (Fig. 4b, c). Among all 129 differentially transcribed *L1Md_Ts* at P5, 126 of them were upregulated in naïve mESCs, in contrast to only three of them upregulated in primed mEpiSCs. Similarly, 74 of 80 differentially expressed *L1Md_Ts* were upregulated in naïve cells at P15, but only 6 were upregulated in primed cells (Fig. 4b; Additional file 4: Table S3; Additional file 5: Table S4). Other retrotransposons, such as *SINEs* including *Alu* family integrant *B1_Mus1*, *B4* family integrant *ID_B1*, and *B2* family integrant *B3*, also were elevated to various degrees in naïve cells (Fig. 4c). *MERVL*, which reportedly is highly expressed in naïve mESCs and can mark 2C-like state [28], was not the most significantly increased retrotransposons in comparison with those of primed cells (Fig. 4c). This might be because *MERVL* is only highly expressed in naïve 2C-like subpopulations, compromising its total expression levels in a large ESC population.

Moreover, all of the top 20 TE families upregulated in primed cells also were retrotransposons (Fig. 4c). Members of the *ERVK* family, including *MMETn-int* and *ETnERV3-int*, and particularly *IAPEz-int*, were transcribed almost exclusively in primed cells (Fig. 4b–d). Among all 46 differentially transcribed *IAPEz-ints*, 43 were upregulated in primed mEpiSCs at P5. This trend was even more obvious in cells at P15, in which 104 of 113 differential *IAPEz-ints* were upregulated in primed cells, and only 9 upregulated in naïve cells (Fig. 4b; Additional file 4: Table S3; Additional file 5: Table S4). Other retrotransposons such as loci of the elements *ID_B1*, *B3* and *B1_Mus1* were upregulated in both naïve and primed cells (Fig. 4b, c).

Immunofluorescence microscopy confirmed that *MuERVL* was sporadically expressed in naïve PSCs but suppressed in primed PSCs (Fig. 4e, f). This result is in accordance with the repression of 2C genes in primed cells (shown above). These data demonstrated highly polarized retrotransposons in naïve and primed PSCs in that naïve mESCs are marked by high transcripts of *L1Md_T* whereas primed mEpiSCs are featured with high transcriptional level of *IAPEz-int*.

### Epigenetic regulation of transcription of naïve and primed PSCs

We explored the underlying mechanisms of how retrotransposons and 2C genes are repressed in primed cells. Initially, we examined the expression pattern of potential histone epigenetic modifications, including H3K9me3, H3K27me3, and H3K27ac, by immunofluorescence microscopy, which revealed no significant difference between naïve and primed cells (Additional file 1: Figure S7e). H3K9me3 exhibited typical foci with DAPI-stained heterochromatin, and H3K27ac and H3K27me3 showed similar nuclear distribution in naïve and primed cells. The expression pattern of PSCs differed greatly from that of differentiated MEF cells served as a control (Additional file 1: Figure S7e).

**Fig. 5:**
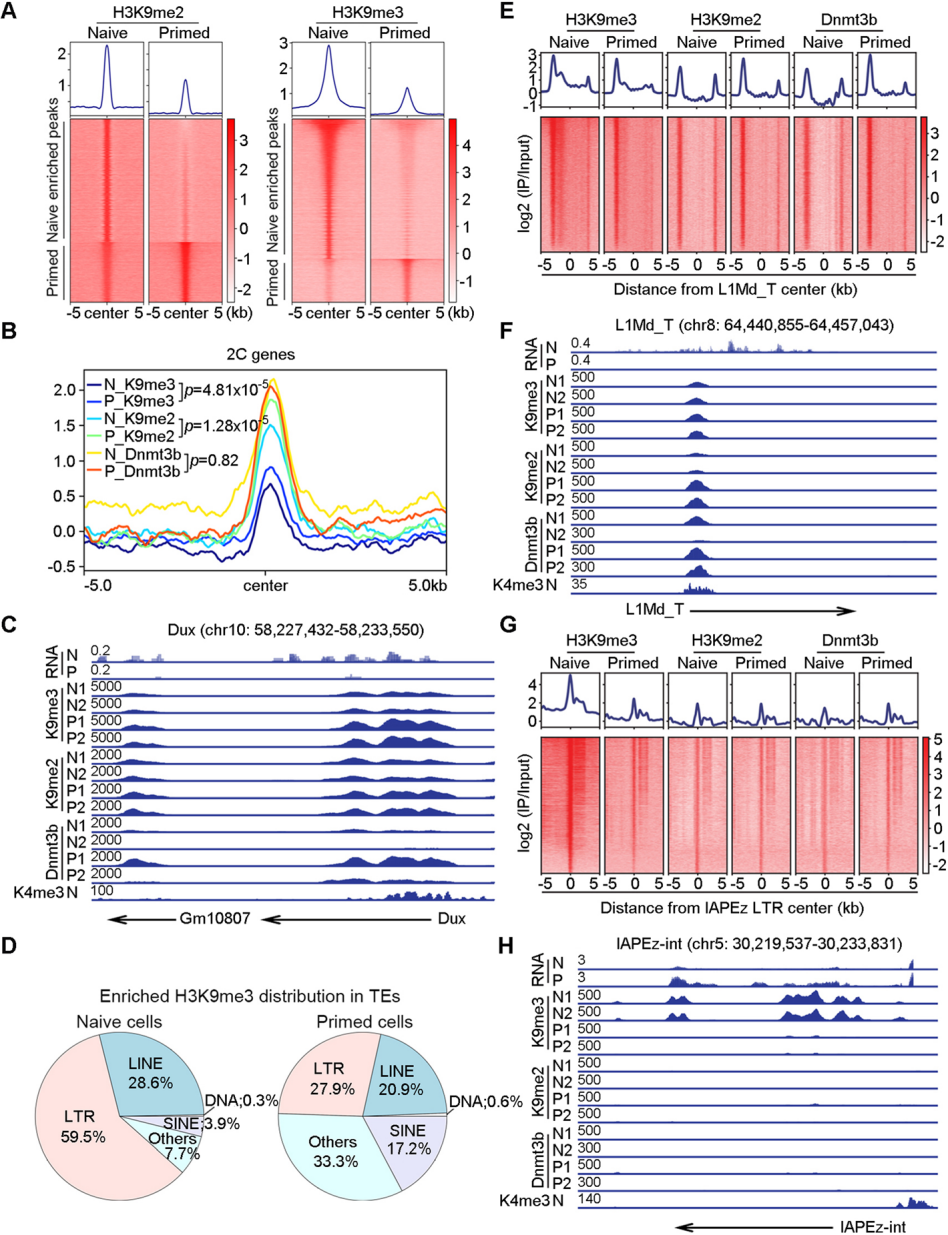
Epigenetic regulation of gene expression and TEs in naïve and primed cells. **A** H3K9me2 (left) and H3K9me3 (right) enrichment signal in differential enriched peaks. There are 28,347 and 32,326 enriched H3K9me2 and H3K9me3 peaks in naïve PSCs and 13,701 and 9,832 enriched H3K9me2 and H3K9me3 peaks in primed PSCs, respectively. The ChIP-seq signals are calculated as log2 ratio of normalized reads relative to the input. **B** H3K9me3, H3K9me2, and Dnmt3b enrichment signal profile plot around the TSS of 2C genes in naïve and primed PSCs, respectively. The ChIP-seq signals are calculated as log2 ratio of normalized reads relative to the input. The Wilcoxon signed-rank test was used to calculate the significance of difference. **C** Density plot of RNA-seq and ChIP-seq signal of *Dux* locus. Arrow indicates the direction of transcription. H3K4me3 was used to indicate the promoters of genes and analyzed based on published data from naïve mESCs cultured in serum/Lif [69]. RNA-seq signals are normalized by CPM, ChIP-seq signals are normalized by RPKM. **D** Differential enriched H3K9me3 peaks distribution in TEs. Peaks located in regions outside of TEs are labeled as Others. **E, G** Plot of H3K9me3, H3K9me2, and Dnmt3b binding profile and heatmap at all *L1Md_T* and *IAPEz-int* loci in naïve and primed PSCs. The ChIP-seq signals are calculated as log2 ratio of normalized reads relative to the input. **F, H** Density plot of RNA-seq and ChIP-seq signal of representative *L1Md_T* and *IAPEz-int* loci in naïve and primed PSCs. Arrow indicates the direction of transcription. H3K4me3 were used to indicate the promoters of genes. RNA-seq signals are normalized by CPM, and ChIP-seq signals are normalized by RPKM

To determine the distribution of histone epigenetic modifications across the genome, we performed bulk chromatin immunoprecipitation coupled with high-throughput sequencing (ChIP-seq) and ultra-low-input micrococcal nuclease-based native ChIP sequencing (ULI-NChIP-seq) assay of H3K9me3, H3K9me2, and Dnmt3b. At the genome-wide level, differential peaks between H3K9me2 and H3K9me3 were mainly enriched in naive cells (Fig. 5a). Firstly, we looked into the enrichment at naïve marker genes such as *Tbx3* and *Klf4* by the ChIP-seq data. Distinctly, H3K9me3 enriched at the promoter region of *Tbx3*, while H3K9me2 and Dnmt3b enriched at the promoter region of *Klf4* in primed cells (Additional file 1: Figure S8a, b), likely repressing their expression, respectively.

Next, we assessed the abundance distribution of Dnmt3b and the two epigenetic modifications H3K9me2 and H3K9me3 across the 2C genes. H3K9me3 and H3K9me2 were more enriched in primed cells across the 2C genes compared to the naïve cells, but no significant difference in Dnmt3b enrichment was found (Fig. 5b). We analyzed the published *Setdb1*, *G9a*, and *Dnmt3b* (H3K9me3, H3K9me1/2, and DNA methyltransferases gene, respectively) KO RNA-seq data in naïve mESCs [70–72]. The naïve genes and 2C genes were upregulated after *Setdb1* and *G9a* KO, while only the naïve genes increased after *Dnmt3b* KO, the 2C genes were not affected (Additional file 1: Figure S8g-l), demonstrating that 2C genes were mainly regulated by H3K9 methylation rather than DNA methylation.

As 2C genes are expressed in only a small percentage (1–5%) of mESC populations, the average abundance signal of the histones could be masked when comparing the whole cell populations in naïve and primed PSCs. Nonetheless, some important genes still manifested differences in the abundance of histones between the two pluripotent states. For instance, *Dux* was discovered as a central transcription regulator of zygotic genome activation in 2C embryos and critical to the expression of *MERVL* and 2C genes during development and in mESCs [73–75]. In our RNA-seq and ChIP-seq data analysis, *Dux* was downregulated in primed mEpiSCs compared to naïve mESCs, and the downregulation was accompanied by elevated levels of H3K9me3, H3K9me2, and Dnmt3b at *Dux* promoter region marked by H3K4me3 (Fig. 5c). These data suggested that H3K9me3, H3K9me2, and Dnmt3b orchestrate in reducing expression of naïve pluripotency genes and suppressing specific 2C genes in primed cells.

### Distinct epigenetic regulation of *L1Md_T* and *IAPEz* in naïve and primed PSCs

The highly expressed specific retrotransposons in naïve and primed PSCs prompted us to understand their molecular regulation by potential epigenetic modifications. Notably, H3K9me3 bound much more LTRs in naïve cells than in primed cells (Fig. 5d), suggesting that H3K9me3 might play a fundamental role in silencing ERVs in naïve pluripotent cells. Then we examined the top two expressed RTEs in naïve and primed cells, the L1 family *L1Md_T* and *L1Md_A*, ERVs *IAPEz* and *ETnERV3*, respectively. As to L1 family, Dnmt3b bound more *L1Md_T* and *L1Md_A* in primed cells compared with those of naïve cells (Fig. 5e; Additional file 1: Figure S8c), indicating that DNA methylation might primarily regulate L1 RTEs in these two pluripotent cells. ChIP-seq data showing enrichment on specific sites of these two L1 families also confirm our conclusion. Especially, Dnmt3b bound more at *L1Md_T* and *L1MT_A* sites in primed cells than in naïve cells; meanwhile, H3K9me2 also seemed to play a repressive role in primed cells (Fig. 5f; Additional file 1: Figure S8d). As to the regulation of *IAPEz* and *ETnERV3*, ChIP-seq data revealed significantly decreased enrichment of H3K9me3 near the TSS site and also across these two ERVs in primed cells (Fig. 5g, h; Additional file 1: Figure S8e, f); thus, this reduced heterochromatic distribution might de-repress *IAPEz* and *ETnERV3*, resulting in their highly specific expression under the primed state. The H3K9me2 and Dnmt3b showed only minimal enrichment on these two ERVs sites (Fig. 5g, h; Additional file 1: Figure S8e, f). These data suggested that diverse epigenetic modifications regulate specific transcription of RTEs in naïve mESCs and primed mEpiSCs. Reduced Dnmt3b (DNA methylation) may promote specific L1 family transcription of *L1Md_T* and *L1Md_A* under naïve state, whereas *IAPEz* and *ETnERV3* transcription is mainly regulated by H3K9me3 in primed PSCs.

Kap1 is a co-repressor in mESCs and tethers to DNA by sequence-specific Kruppel-associated box zinc finger proteins (KRAB-ZFPs) and induces local heterochromatin formation through the histone methyltransferase Setdb1, responsible for H3K9me3, and has been shown to control endogenous retroviruses in ESCs [38, 39]. Our RNA-seq data and western blot analysis showed that Kap1 was downregulated in primed cells compared to naïve cells (Additional file 1: Figure S9a). We hypothesized that decreased Kap1 could lead to upregulation of *IAPEz-int* in primed cells. We analyzed the published sequencing data of *Kap1* knockdown (KD) ESCs [75, 76]. *IAPEz-int* was the most significantly upregulated type of retrotransposons (Additional file 1: Figure S 9b, c). The enrichment of Kap1 was very high at the *IAPEz-int* loci in ESCs and the enrichment of H3K9me3 at the *IAPEz-int* site was dramatically decreased after *Kap1* KD (Additional file 1: Figure S9d, e, g). Likewise, Kap1-mediated H3K9me3 also has an inhibitory effect on *L1Md_T* (Additional file 1: Figure S 9d, f, g). These data together further support the notion that heterochromatic histone modifiers can regulate *IAPEz-int* and *L1Md_T* transcription in PSCs.

### Primed PSCs manifest increased genomic instability in association with retrotransposon insertion

**Fig. 6:**
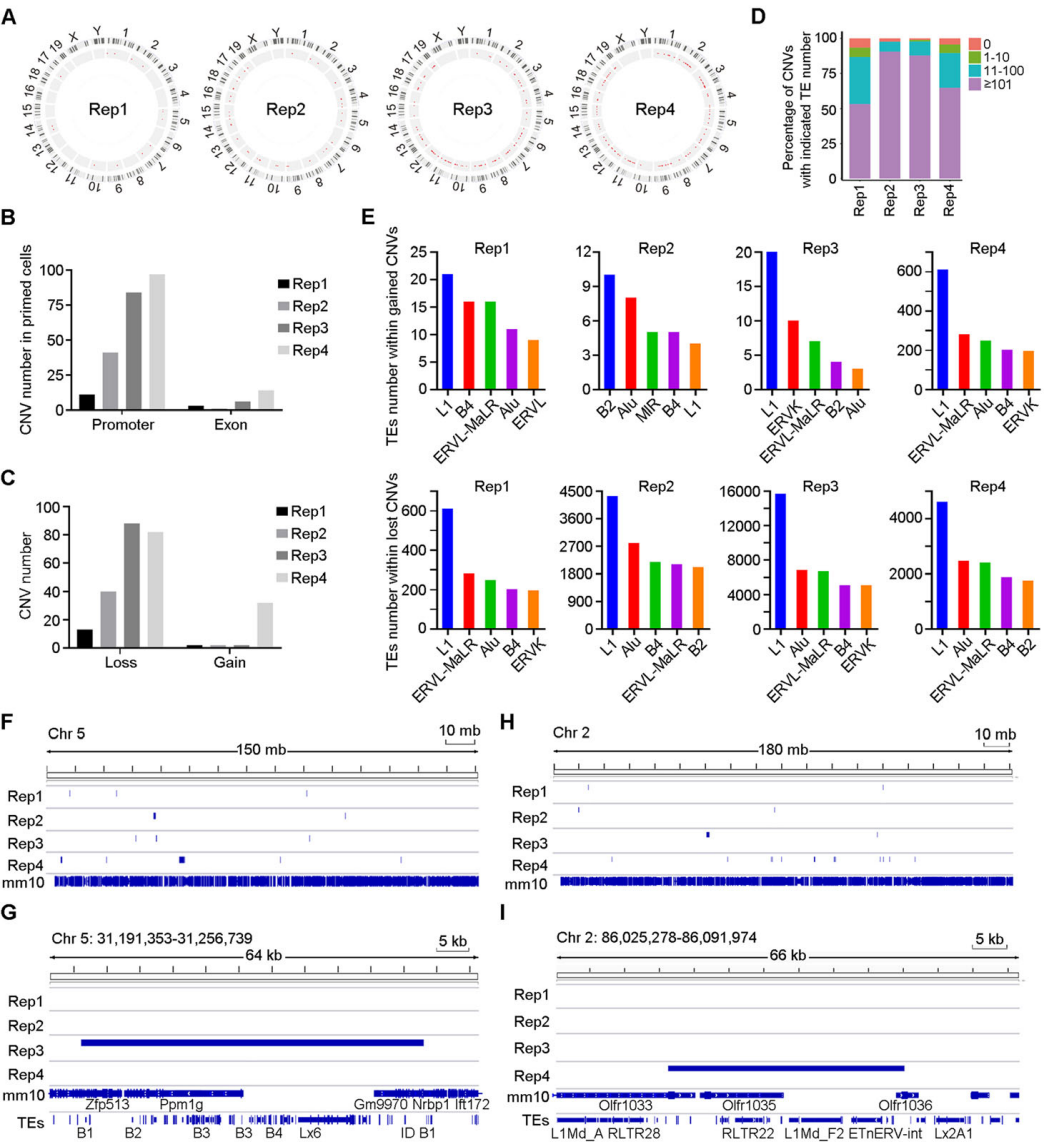
Primed mEpiSCs harbor more CNVs than do naïve mESCs. **A** Circos plot showing genetic alterations in primed cells from CBA × C57 genetic background. The red circle represents CNVs. n = 4 different cell line clones. **B** Genome distribution of CNVs in primed cells. **C** Number of gained and lost CNVs in primed cells. **D** Percentage of CNVs with different TE numbers. Four classes of CNVs were classified according to the number of overlapped TEs. **E** Number of TEs in the top five families contained within gained or lost CNVs sequence. **F** Genome browser view of the distribution of CNVs identified in chromosome 5 in primed cells. Blue markers represent gained CNVs. **G** Genome browser view by IGV of the distribution of gained CNVs identified in chromosome 5 mapping with TEs in primed cells. **H** Genome browser view of the distribution of CNVs identified in chromosome 2 in primed cells. Blue markers represent gained CNVs. **I** Genome browser view by IGV of the distribution of gained CNVs identified in chromosome 2 mapping with TEs in primed cells. All n = 4

Lastly, we were curious to know any consequences, if any, resulting from differential DNA repair capacity and expression of retrotransposons in naïve versus primed PSCs. We compared the genomic stability of seven different clones from two different genetic background cell lines under naïve and primed state by conducting the whole-exome sequencing (WES) analysis. We applied the naïve ESCs served as references and compared the primed with naïve ESCs, because the naïve ESCs efficiently produced germline chimeras as well as TEC pups, indicating that the naïve ESCs are genomic stable. By such comparison with naïve ESCs, any insertions and the TEs contained within copy number variations (CNVs) in primed ESCs presumably are considered as de novo and specific. Six out of seven primed cell line clones from two genetic backgrounds had more CNVs compared to the naïve clones, and these CNVs contained similar families of TEs like *L1*, *Alu*, *B2*, and *B4*. The variable number of CNVs and TEs within CNVs indicates the heterogeneity of different clones (Additional file 6: Table S5). Notably, primed cells in CBA × C57 background accumulated a higher number of CNVs compared to naïve cells (Fig. 6a). Among the four cell line clones at CBA × C57 background, CNVs of primed cells mainly were found in the promoter regions (Fig. 6b). Most of them showed lost CNVs (Fig. 6c). Remarkably, when all the lost and gained CNVs in primed cells were mapped to the genome, most of CNVs contained more than 100 TEs (Fig. 6d) of various families, especially *L1*, *Alu*, and *ERVK* (Fig. 6e). As examples, the CNVs occurred on chromosomes 2 and 5 and the TEs within CNVs were found variable in different clones (Fig. 6f–i). Similar observation was also found in another cell line at 129 × C57 background and again TEs contained in CNVs were variable (Additional file 6: Table S5). These results demonstrated increased genomic instability in the primed ES cells and suggested that more CNVs in primed ES cells could be introduced by insertion of aberrantly activated retrotransposons.

## Discussion

**Fig. 7:**
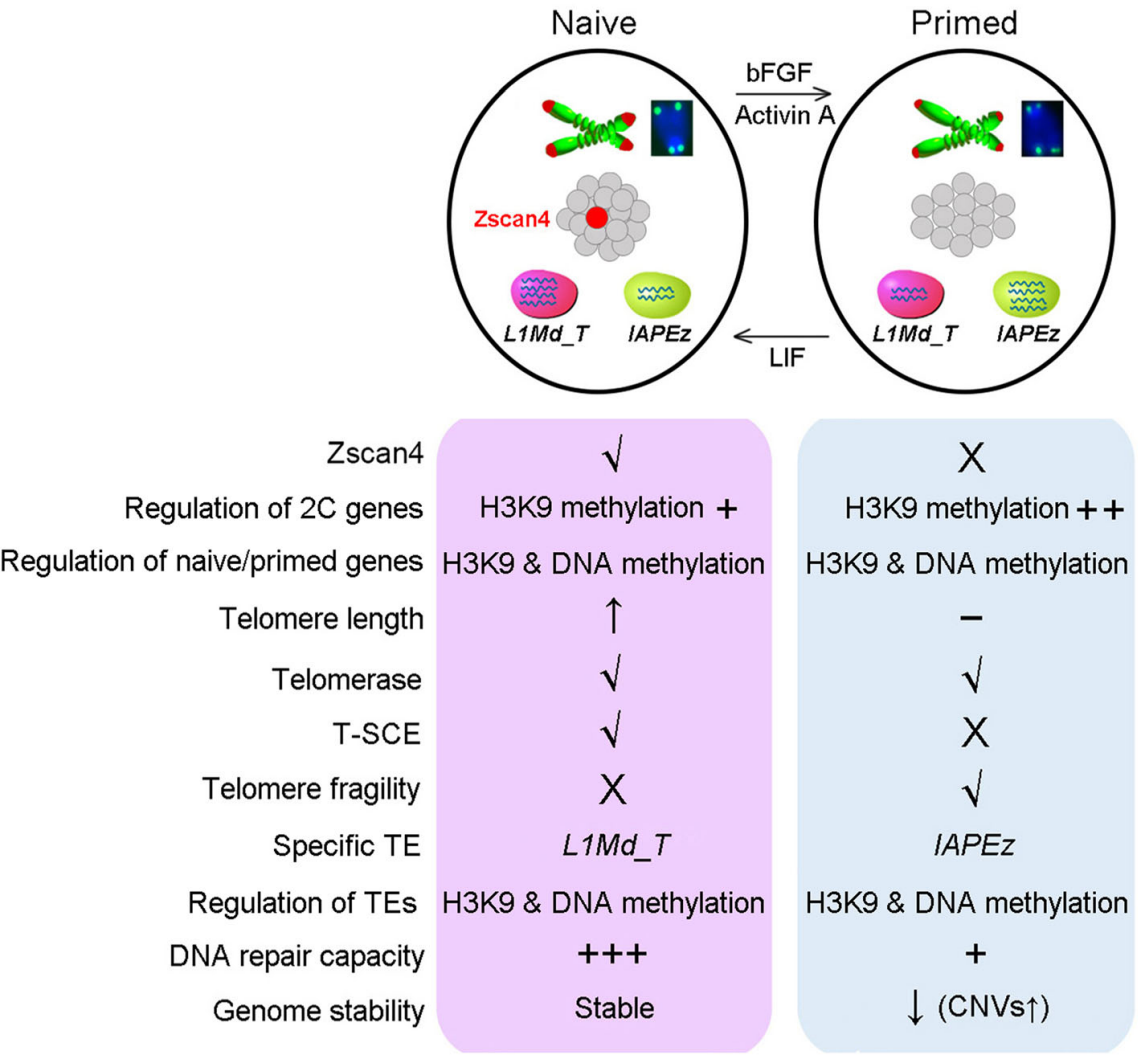
Schematic diagram showing distinct telomere regulation and retrotransposon transcription between naïve and primed cells. Telomeres lengthen significantly by telomerase and ALT pathway in naïve cells, which is associated with activation of the 2C genes and particularly sporadic expression of *Zscan4*. Repression of 2C genes including *Dux* in primed state cells is mainly caused by H3K9 methylation. While telomere elongation cannot be achieved by telomerase alone, telomere fragility occurs in primed cells, in association with decreased DNA recombination repair capacity. Naïve PSCs utilize both telomerase and telomere mechanisms to elongate telomeres, whereas primed PSCs have only telomerase to maintain telomeres. Nevertheless, the telomerase is insufficient after longer cultures of primed PSCs such that telomeres become shortened. Diverse inhibitory epigenetic modifications act together to regulate the retrotransposon transcription, resulting in specific expression of *L1Md_T* and *IAPEz-int* in naïve and primed cells, respectively. Moreover, decreased DNA repair capacity, aberrant transcription and insertion of retrotransposons are linked to increased genome instability in primed PSCs

We find that primed PSCs exhibit fragile telomere with passages and retrotransposon regulation by various histone modifications at specific loci, and deficient DNA recombination repair, distinct from naïve PSCs. Consequently, primed PSCs harbor increased frequency with passages of CNVs and genomic instability where retrotransposon integration is found, in contrast to naïve PSCs (Fig. 7). Our naïve ESCs were maintained on the presence of feeder cells under conventional serum/LIF culture conditions and exhibited developmental pluripotency as evidenced by efficient generation of germline chimeras as well as TEC pups that lived healthily to adulthood and reproduced, also indicating that the naïve ESCs were genomic stable.

Primed PSCs show reduced DNA recombination and repair capacity as well as low mismatch repair and nucleotide-excision repair capacity, different from naïve PSCs. DSB repair process is very rapid and homologous recombination-mediated repair (HRR) may predominate in mESCs [77]. *Rad51* and *Brca1*, involved in HRR [78], are downregulated under primed state. The high DNA recombination and repair capacity are important for maintaining telomere function and genomic stability in naïve cells. Telomeres lengthen under naïve state whereas elongation of telomeres is suppressed in primed cells, and this is linked to the repression of 2C genes and particularly *Zscan4* that is critical for telomere lengthening and maintaining the genomic stability of mESCs by T-SCE-based homologous recombination [52, 79]. *Zscan4* repression for longer term can impair naïve pluripotency and developmental potential of mESCs in vivo [25] and induce expression of differentiation-related genes [80]. T-SCE also is suppressed and high telomerase activity is required for telomere elongation in conventional primed human ESCs [81]. Lack of telomere recombination-induced telomere elongation corroborates increased frequency of fragile telomeres found in primed PSCs at later passage. The fragile sites are unstable under replication stress, and the fragile telomeres, as a result of replication stress at telomeres, would be more likely to result in chromosome instability due to deficiency in DSB repair at subtelomeric regions [54, 82]. Impaired telomere elongation by the accumulation of repressive histones and declined capacity of DNA recombination repair in association with fragile telomeres may lead to genomic instability of primed cells. In consistency, telomere erosion in human pluripotent stem cells conventionally known as primed state of pluripotency induces DNA damage response and leads to ATR-mediated mitotic catastrophe [83]. Also, mESCs depleted for telomere maintenance protein *TRF2* elicit minimal or only mild DNA damage response, in contrast to non-pluripotent or differentiated cells showing that TRF2-mediated telomere protection is dispensable in pluripotent stem cells [84, 85]. Notably, *TRF2* depletion in mESCs activates a totipotent-like two-cell-stage transcriptional profile including high levels of *Zscan4* and the upregulation of *Zscan4* reduces DNA damage and elongates telomeres in the absence of TRF2 [85], providing additional evidence in supporting importance of Zscan4 in pluripotent stem cells.

Increasing evidence established that Zscan4 is critical to telomere elongation and maintenance of naïve mouse ESCs. However, mechanisms underlying Zscan4-mediated telomere elongation remain elusive. Telomeres shortened after *Dmc1* KO in naïve ESCs and slightly shortened after *Rad51* KO (Additional file 1: Figure S6e, f), implying that DNA recombination is involved in telomere maintenance. We have attempted to exogenously overexpress *Dmc1* in the *Dmc1* KO naïve cell lines but failed in achieving the *Dmc1* rescue cells. As we did not overexpress *Dmc1* in WT naïve cells, we cannot conclude whether overexpression of *Dmc1* is toxic to the cells or whether this phenotype is an off-target effect. Also, it is likely that the telomere phenotype in primed state cells may be caused by comprehensive factors. Overexpression of a single factor alone may not be sufficient to rescue telomere phenotype in primed state cells. It will be necessary to systematically investigate telomere function and regulation in naïve and primed PSCs in future experiments.

Moreover, differential transcription of retrotransposons clearly distinguishes mouse primed from naïve states. *L1Md_T* is highly transcribed in naïve ESCs and *IAPEz-int* in primed cells. LINE1 regulates global chromatin accessibility in the early mouse embryo and also acts as a repressor of *MERVL* to balance 2-cell-like activity in mESCs [86, 87]. These data also support the notion that *L1* contributes to the pluripotency network [86]. Indeed, we also observed higher expression levels of *L1* than those of *MERVL* in naïve PSCs. Expression of retrotransposons is known to be regulated by a variety of epigenetic modifications, including DNA methylation and histone methylation [88–90]. Retrotransposons in naïve and primed cells are differentially regulated by diverse epigenetic modifications. For example, downregulation of *L1Md_T* in the primed state is mainly regulated by DNA methylation, and transcription of *IAPEz-int* is mainly regulated by histone methylation, such as H3K9me3. In support, heterochromatin modifier SETDB1 prevents TET2-dependent activation of *IAP* retroelements (especially *IAPEz-int*) in mESCs [91]. Diverse epigenetic modifications also regulate *Dux* expression, which in turn can activate downstream 2C genes such as *Zscan4* and *MERVL* that serve as enhancers of 2C genes including *Zscan4* itself [73–75, 80]. *Zscan4*, in turn, activates *MERVL* and cleavage embryo genes [80]. In naïve PSCs, the highly expressed *Dux* activates a series of downstream 2C genes, including *Zscan4*, which can activate *MERVL* and rapidly lengthens telomeres through T-SCE. A large amount of *LINE1* transcription is required to inhibit excessive 2C gene expression to maintain genome stability and pluripotency of naïve cells. In contrast, under primed state, diverse inhibitory heterochromatin epigenetics act in combination at the *Dux* gene promoter region to inhibit *Dux* expression and further repress expression of downstream 2C genes. Therefore, the primed cells do not need *LINE1* to inhibit 2C genes. Recently, *Dux* expression is reported to facilitate nuclear transfer and somatic reprogramming by activating downstream 2C genes and plays an important role during 2C-like to pluripotent state transition process [92, 93].

However, aberrant *L1* transcripts that have retrotransposition activity may harm the host genome by random integration and consequently lead to genomic instability [94], even though fewer *L1* is activated in primed cells than in naïve cells. Indeed, exome-seq data reveals that primed cells accumulate more CNVs containing various TEs. Although a relatively large CNV contains one or many TEs, the CNV boundaries do not necessarily indicate the mechanism underlying the induction of CNV by TEs. RNA-seq data shows that primed ESCs express genes for lineage specification such as *Eomes* and *Gsc* as mesendoderm marker genes [95], suggestive of differentiation properties. Additionally, primed ESCs exhibit reduced DNA repair capacity, increased telomere fragility, declined mitochondria functions, and aberrant cell cycle. These factors together could compromise developmental potential of primed ESCs.

## Conclusions

Primed PSCs have been known to have lower pluripotency than do naïve PSCs. Our data reveals that primed PSCs in mice exhibit declined telomere maintenance and elevated retrotransposon-associated genomic instability, coincided with reduced DNA recombination repair capacity. Specifically, (1) Telomeres lengthen in naïve PSCs, which acquire robust recombination repair, but not in primed state, such that naïve PSCs maintain telomere integrity, whereas primed PSCs exhibit fragile telomeres; (2) Naïve and primed states show distinct retrotransposon activation and related epigenetic state; (3) Genomic instability is increased in primed PSCs, in association with the insertion of retrotransposons and increased CNVs in contrast to naïve PSCs; (4) Reduced function in cell cycle and mitochondria and differentiation properties are found in primed PSCs. Mitochondria dysfunction has been linked to telomere attrition. Fragile telomeres, decreased DNA recombination repair capacity, and aberrant retrotransposon activity together likely contribute to increased genomic instability found in primed mouse PSCs after prolonged culture in vitro. This also may explain why mouse naïve PSCs achieve developmental pluripotency by stringent functional assays, but primed PSCs do not. These findings could have implications in further derivation and characterization of human naïve and primed PSCs.

## Methods

### Animal care and use

Use of mice for this research was approved by the Nankai University Animal Care and Use Committee. All mice used in this study were taken care of and operated according to the relevant regulations. Mice were housed and cared in individually ventilated cages (IVCs) on a standard 12 h light: 12 h dark cycle in the sterile Animal Facility. Oct4-GFP (OG2) mice (CBA × C57, JAX stock #004654) that carry Oct4 distal promoter-driven GFP were purchased from Model Animal Research Center of Nanjing University. 129 × C57 mice, albino ICR mice, and albino Kunming (KM) mice were purchased from Beijing Vital River Laboratory Animal Technology Co., Ltd. The mice were humanely euthanized for specific experiments.

### Derivation of naïve mESCs

Conventional naïve mESC lines were established and characterized based on the method described and reviewed previously [96]. Blastocysts were isolated from the uterine horns of pregnant females at embryo (E) 3.5 using a dissection microscope in HKSOM and plated onto mitomycin C-treated MEF cells served as feeders in KSR/DMEM (K/DL) medium and cultured for 7 days to form outgrowths. Emerging ICM outgrowths were directly picked into serum/LIF (S/L) medium on feeders to establish stable naïve mESC lines. mESCs were maintained by dissociating cells with 0.25% TE every 2–3 days and re-plating them onto feeder cells. K/DL medium contains knockout DMEM (Invitrogen) supplemented with 20% Knockout serum replacement (KSR, Invitrogen), 1 mM l-glutamine, 100 μM NEAA, 1% 2A, 0.1 mM β-mercaptoethanol (β-me, Invitrogen), 1 μM PD0325901 (Miltenyi), and 1000 IU/ml mouse LIF (mLIF, Millipore). S/L medium (ESC culture medium) contains knockout DMEM supplemented with 20% FBS (ES quality, Hyclone), 1 mM l-glutamine, 100 μM NEAA, 1% 2A, 0.1 mM β-me, and 1000 IU/ml mLIF.

### ESC cultures

Three mouse ES cell lines were used in this study. OG2 ESC line was derived from C57BL6 × CBA mice that carry *Oct4* distal promoter-driven GFP, 129 × C57 ESC line from B6 × 129F1 mice, and AKJ2 ESC line from actin-GFP mice in C57BL/6J origin. Naïve mESCs were routinely cultured based on the method described previously [23]. Briefly, mESCs were cultured under 5% CO_2_ at 37 °C on mitomycin C-treated MEF feeder in S/L medium consisting of knockout DMEM supplemented with 20% fetal bovine serum (FBS, ES quality, Hyclone), 1000 U/ml leukemia inhibitory factor (LIF) (ESGRO, Chemicon), 0.1 mM non-essential amino acids, 0.1 mM β-mercaptoethanol, 1 mM l-glutamine, and penicillin (100 U/ml) and streptomycin (100 μg/ml). Naïve mESCs were maintained by dissociating cells with 0.25% TE every 2–3 days and re-plating them onto feeder cells.

Conversion of mEpiSCs from naïve mESCs was achieved based on the method described [11]. In brief, mESCs were seeded at a density of 10^5^ cells per 6-well plate in S/L medium on feeder cells. On the next day, the medium was replaced with mEpiSCs bFGF/Activin A (F/A) medium. After culture for 3–4 days, the surviving cells grew and formed large compact colonies. These primed EpiSCs were passaged every 3–4 days on feeder cells by collagenase IV (1 mg/ml; Invitrogen) dissociated as cell clumps or mechanical dissociation by syringe needles. The F/A medium contains a 1:1 mixture of DMEM/F12 (Invitrogen) supplemented with N2 (Invitrogen) and Neurobasal (Invitrogen) supplemented with B27 (Invitrogen), 1 mM l-glutamine, 0.1 mM non-essential amino acids, penicillin (100 U/ml) and streptomycin (100 μg/ml), 0.1 mM β-mercaptoethanol, 1% KSR, 12 ng/ml bFGF (Invitrogen), and 20 ng/ml Activin A (Peprotech). The density of feeder cells was crucial to maintaining these mEpiSCs passaging in vitro in an undifferentiated state.

### Production of chimeras and genotyping

Approximately 10–15 naïve mESCs or primed mEpiSCs were injected into 4–8-cell embryos of ICR mice as hosts using a Piezo injector as described [96]. Injected embryos were cultured overnight in KSOM_AA_ medium. Blastocysts were transferred into uterine horns of E2.5 surrogate mice. Pregnant females delivered pups naturally at about E19.5. Pups were identified initially by coat color. Contribution of mESCs or mEpiSCs to various tissues in chimeras was confirmed by standard DNA microsatellite genotyping analysis using *D12Mit136* primers: 5′-TTA ATT TTG AGT GGG TTT GGC-3′ and 5′-TTG CTA CAT GTA CAC TGA TCT CCA-3′.

### Tetraploid embryo complementation (TEC)

To generate mice by tetraploid embryo complementation, two-cell embryos were collected from the oviducts of ICR females and electrofused to produce one-cell tetraploid embryos that were then cultured in KSOM media. Naïve mESCs were injected into the tetraploid blastocyst cavity. The blastocysts were placed in KSOM with amino acids until embryo transfer. Approximately thirty injected blastocysts were transferred to each uterine horn of 2.5-day-postcoitum pseudopregnant ICR females. Pregnant recipients with tetraploid embryos were subjected to cesarean section on day 18.5 of gestation. TEC mice further were mated with ICR mice to test their fertile capacity. The contribution of naïve mESCs to TEC mice was confirmed by standard DNA microsatellite genotyping analysis using *D12Mit136* and *D12Mit99* primers (5′- CTT ACA GAA AAT GAA AAC CAA AAC A-3′ and 5′-CCT CTG CTT TAG AGG CAA ACG-3′).

### DNA damage response

To induce DNA damage, naïve and primed PSCs were exposed to 2.5 μM etoposide or 5 mM H_2_O_2_ for 2 h. Two hours and 20 h following the exposure, cells were collected for immunofluorescence microscopy of 53BP1 or γH2AX foci, commonly used as DNA damage response markers [53]. More than 50 cells were counted for quantification of each group.

### Immunofluorescence microscopy

Cells were washed twice in PBS, fixed in freshly prepared 3.7% paraformaldehyde for 30 min on ice, washed once in PBS, and permeabilized in 0.1% Triton X-100 in blocking solution (3% goat serum plus 0.1% BSA in PBS) for 30 min at room temperature, then washed once in PBS, and left in blocking solution for 2 h. Cells were incubated overnight at 4 °C with primary antibodies anti-Oct4, Nanog, SSEA1, Dnmt3b, 53BP1, γH2AX, or Zscan4. Cells were washed three times with blocking solution and incubated for 1 h with secondary antibodies at room temperature. Goat Anti-Mouse IgG (H + L) FITC, Goat Anti-Rabbit IgG (H + L) Alexa Fluor® 594, and Donkey Anti-Goat IgG (H + L) Alexa Fluor® 594 diluted 1:300 with blocking solution were used. Samples were washed and counterstained with 0.5 μg/ml DAPI (Roche) in Vectashield (Vector) mounting medium. Fluorescence was detected and imaged using Axio-Imager Z2 Fluorescence Microscope (Carl Zeiss).

### Western blot

Cells were washed twice in PBS, collected, and lysed in cell lysis buffer on ice for 30 min and then sonicated for 1 min at 60 of amplitude with 2-s intervals. After centrifugation at 10,000*g*, 4 °C for 10 min, supernatant was transferred into new tubes. The protein concentration was measured by bicinchoninic acid, and then protein samples were boiled in SDS Sample Buffer at 95 °C for 10 min. Same amount of protein of each cell extract was resolved by 10% Acr-Bis SDS-PAGE and transferred to polyvinylidene difluoride membranes (PVDF, Millipore). Nonspecific binding was blocked by incubation in 5% skim milk in TBST at room temperature for 2 h. Blots were then probed with primary antibodies overnight by incubation at 4 °C with Oct4, Nanog, Dnmt3b, or Zscan4, and β-actin served as a loading control. Immunoreactivity bands were then probed for 2 h at room temperature with the appropriate horseradish peroxidase (HRP)-conjugated secondary antibodies, goat anti-Rabbit IgG-HRP, or goat anti-Mouse IgG (H + L)/HRP. Protein bands were detected by chemiluminescent HRP substrate (WBKLS0500, Millipore). Information of antibodies is listed in Additional file 7: Table S6.

### Dot blot

Total 5mC and 5hmC levels were measured by dot blot analysis based on the method described [97]. Briefly, genomic DNA was denatured in 0.4 N NaOH and 10 mM EDTA at 95 °C for 10 min, and 2-fold serial dilutions were spotted on positively charged nylon membranes. The membranes were immunoblotted with 5mC and 5hmC antibody, followed by HRP-conjugated goat anti-mouse or anti-rat antibodies. DNA loading was verified by staining with methylene blue.

### Generation of *Dmc1*, *Rad51*, or *Brca1* KO naïve mESCs by CRISPR/Cas9

Briefly, CRISPR/Cas9 expression vector pSpCas9(BB)-2A-Puro (PX459) was digested with BbsI endonuclease (Fermentas). The sgRNA targeting *Dmc1*, *Rad51*, or *Brca1* was designed using http://crispor.tefor.net/crispor.py. sgRNA-F and sgRNA-R were annealed into a double strand as 95 °C for 30 s, 72 °C for 2 min, 56 °C for 2 min, 37 °C for 2 min, 25 °C for 2 min, and 4 °C for storage. Annealed double strand was diluted to 100-fold and constructed to a linearized vector. Positive clones were picked for sequencing, and the sequencing primers were Human U6 Promoter-F (ACT ATC ATA TGC TTA CCG TAA C). After sequencing, the correct bacterial solution was chosen for expansion culture and the ultrapure plasmid extracted for cell transfection. After transfection, cells were screened by puromycin and then selected and expanded from a single clone. The knockout cells were characterized by PCR and sequencing. The knockout cells were cultured for additional 8 passages prior to measurement of telomeres. The sequences of sgRNA and PCR detection primers are listed in Additional file 7: Table S6.

### Telomerase activity by TRAP assay

Telomerase activity was determined by the Stretch PCR method according to manufacturer’s instruction using TeloChaser Telomerase assay kit (T0001, MD Biotechnology). About 2.5 × 10^4^ cells from each sample were lysed. Lysis buffer served as negative controls. PCR products of cell lysate were separated on non-denaturing TBE-based 12% polyacrylamide gel electrophoresis and visualized by ethidium bromide (EB) staining.

### Telomerase activity by ELISA assay

Telomerase activity was quantified using ELISA Kit (CSB-E08022m, CUSABIO) according to the manufacturer’s protocol. About 1 × 10^6^ cells in 300 μl PBS were used for telomerase quantification, and 100 μl standard, blank, or sample added per well, covered with the adhesive strip and incubated for 2 h at 37 °C. Biotinantibody working solution (100 μl) was added to each well and incubated for 1 h at 37 °C. After washing for three times, 100 μl HRP-avidin working solution was added to each well, covered with a new adhesive strip, and incubated for 1 h at 37 °C. After washing, 90 μl TMB substrate was added to each well and incubated for 15–30 min at 37 °C. The optical density of each well was determined using a microplate reader set to 450 nm.

### Telomere restriction fragment (TRF) measurement

TRF analysis was performed using a commercial kit (TeloTAGGG Telomere Length Assay, cat no. 12209136001, Roche). DNA was extracted from cells by phenol-chloroform method. A total of 3 μg DNA was digested overnight with MboI endonuclease (NEB) at 37 °C and electrophoresed through 1% agarose gels in 0.5 × TBE at 14 °C for 16 h at 6 V/cm with an initial pulse time of 1 s and end in 12 s using a CHEF Mapper pulsed-field electrophoresis system (Bio-Rad). The gel was blotted and probed using reagents in the kit. Telomere length is quantified by TeloTool software.

### Telomere measurement by real-time qPCR

Genome DNA was prepared using DNeasy Blood & Tissue Kit (QIAGEN, Valencia, CA). Average telomere length was measured from total genomic DNA using a real-time PCR assay [98]. PCR reactions were performed on the iCycler iQ5 2.0 Standard Edition Optical System (Bio-Rad, Hercules, CA), using telomeric primers, primers for the reference control gene (mouse 36B4 single-copy gene), and PCR settings as described [99]. For each PCR reaction, a standard curve was made by serial dilutions of known amounts of DNA. The telomere signal was normalized to the signal from the single-copy gene to generate a T/S ratio indicative of relative telomere length. Equal amount of DNA (20 ng) was used for each reaction. 36B4 primer: 5′-ACT GGT CTA GGA CCC GAG AAG-3′ and 5′-TCA ATG GTG CCT CTG GAG ATT-3′; Tel primer: 5′-CGG TTT GTT TGG GTT TGG GTT TGG GTT TGG GTT TGG GTT-3′ and 5′-GGC TTG CCT TAC CCT TAC CCT TAC CCT TAC CCT TAC CCT-3′.

### Telomere Q-FISH

Telomere length was estimated by telomere Q-FISH as described [100]. Telomeres were denatured at 80 °C for 3 min and hybridized with FITC-labeled (CCCTAA)_3_ peptide nucleic acid (PNA) probe at 0.5 μg/ml (F1001, Panagene, Korea). Chromosomes were stained with 0.5 μg/ml DAPI. Fluorescence from chromosomes and telomeres was digitally imaged on Imager Z2 Zeiss microscope with FITC/DAPI using AxioCam and AxioVision software 4.6. Telomere length shown as telomere fluorescence intensity was integrated using the TFL-TELO program (kindly provided by Peter Lansdorp, Terry Fox Laboratory). More than 15 metaphase spreads were counted for each group.

### Telomere chromosome orientation-fluorescence in situ hybridization (CO-FISH)

CO-FISH assay was performed as described [59], with slight modification. mESCs or mEpiSCs were incubated with BrdU (10 μM) for 12 h. Nocodazole at 0.3 μg/ml was added for 3 h prior to cell harvest, and metaphase spreads were prepared by a routine method. Chromosome slides were treated with RNase A, fixed with 4% formaldehyde, then stained with Hoechst 33258 (0.5 mg/ml), incubated in 2 × SSC (Invitrogen) for 15 min, and exposed to 365 nm UV light (Stratalinker 1800UV irradiator) for 40 min. BrdU-substituted DNA was digested with Exonuclease III (Takara). Slides were then dehydrated through ethanol series and air-dried. PNA-FISH was performed with FITC-OO-(CCCTAA)_3_ (Panagene, F1009). Slides were hybridized, washed, dehydrated, mounted, and counterstained with 1.25 μg/ml DAPI in VectaShield antifade medium. Digital images were captured using CCD camera on Zeiss Imager Z2 microscope. To analyze telomere sister chromatid exchange (T-SCE), one signal at each end of the chromosome was counted as no T-SCE, while two signals at both chromatids on one chromosome end were identified as one T-SCE. At least 15 metaphase spreads were counted for the frequency of T-SCE. Pairwise comparisons for statistical significance were made by t-tests.

### Metabolic flux analysis

Agilent Seahorse XFe24 Analyzers was used to measure oxygen consumption rate of naïve and primed state PSCs. Cells were plated in XF24 Cell Culture Microplates pre-coated with 1% Matrigel at a density of 60,000 per well. The next day, cells were treated with Seahorse XF Cell Mito Stress Test Kit (10 μg/ml oligomycin, 1 μM FCCP, and 1 μM rotenone and antimycin) and measured following the manufacturer’s instructions.

### Library preparation and RNA-sequencing

mRNA was purified from total RNA using poly-T oligo-attached magnetic beads. Fragmentation was carried out using divalent cations under elevated temperature in NEB Next First-Strand Synthesis Reaction Buffer (5x). First-strand cDNA was synthesized using random hexamer primer and M-MLV Reverse Transcriptase (RNase H^-^). Second-strand cDNA synthesis was subsequently performed using DNA Polymerase I and RNase H. Remaining overhangs were converted into blunt ends via exonuclease/polymerase activities. After adenylation of 3′ ends of DNA fragments, NEB Next Adaptors with hairpin loop structure were ligated to prepare for hybridization. To select cDNA fragments of preferentially 150~200 bp in length, the library fragments were purified with AMPure XP system (Beckman Coulter, Beverly, USA). Then 3 μl USER Enzyme (NEB, USA) was used with size-selected and cDNA adaptor-ligated at 37 °C for 15 min followed by 5 min at 95 °C followed by PCR. PCR was performed with Phusion High-Fidelity DNA polymerase, Universal PCR primers, and Index Primer. At last, PCR products were purified using AMPure XP system and library quality assessed on the Agilent Bioanalyzer 2100 system. Cluster of the index-coded samples was performed on a cBot Cluster Generation System using TruSeq PE Cluster Kit (Illumina) according to the manufacturer’s instructions. After cluster generation, the library preparations were sequenced on an Illumina Hiseq platform.

### Bioinformatics analysis for differentially expressed genes

The adapter sequences of RNA-seq data and low-quality reads with Phred score < 5 were deleted with Cutadapt before further processing. Hisat2 was used to map RNA-seq data to mm10 with parameter - k 20. Genes were annotated according to the Ensembl database. Transposable element annotations were from UCSC Genome Browser (RepeatMasker). Reads were counted using featureCounts. TE transcripts with parameter “–mode multi” was used to measure differentially expressed ERVs. Genes with expression fold change > 2 and adjusted *P* value < 0.01 according to DEseq2 were used for GO and KEGG analysis by clusterProfiler**.** Clustering and analysis of 2-cell embryo, naïve, and primed pluripotent-specific genes were done according to published RNA-seq data [2, 28]. Z-score of selected genes was used for heatmap. DEGs related to cell cycle and apoptotic signaling pathway (downloaded from https://www.gsea-msigdb.org) were plotted in heatmaps.

### Bioinformatics analysis of transcription of transposable elements (TEs)

To precisely estimate the expression of single TE locus, we used the recently published SQuIRE method with “total” mode [68]. SQuIRE function “squire Fetch” was used to download TE annotation from RepeatMasker, then “squire Clean” was used to filter RepeatMasker file for repeats of interest, collapses overlapping repeats. Function “squire Map” was used to align reads to mm10, then “squire Count” was used to quantify reads aligning to TEs and genes. For the TE subfamily level, we add up all count of the TE loci. Only quantification of TE was used by DESeq2 to calculate the differentially expressed TE locus. TE locus whose foldchange > 1.5 and *P* value < 0.01 were considered significant.

### Bulk chromatin immunoprecipitation coupled with high-throughput sequencing (ChIP-seq) data analysis

Bulk ChIP experiment was performed based on the method described [80]. Briefly, naïve and primed PSCs were collected after removing off feeders and fixed with 1% paraformaldehyde, lysed, and sonicated to achieve the majority of DNA fragments with 100–1000 bp. DNA fragments were enriched by immunoprecipitation with 5 μg antibody and dynabeads M280 (Life Technologies). The immunoprecipitated material was eluted from the beads by heating for 30 min at 68 °C. To reverse the crosslinks, samples were incubated with Proteinase K at 42 °C for 2 h followed by 67 °C for 6 h. The samples were then extracted with phenol:chloroform:isoamyl alcohol (25:24:1, pH > 7.8) followed by chloroform, ethanol precipitated in the presence of glycogen, and re-suspended in TE buffer. The adapter sequences and low-quality base were removed by Cutadapt. Bowtie2 was used to map reads to mm10. ChIP-seq signal enrichment was analyzed and obtained by bamCompare from Deeptools [101, 102]. ChIP signal heatmap and line plot were also generated by computeMatrix and plotHeatmap form Deeptools. For gene locus visualization, duplicated reads were marked by MarkDuplicates from picard and removed by samtools. Differential enriched peaks were called by sicer and peaks with log10 of FDR>10 were kept. To analyze the enrichment signal on TE subfamily by ChIP-seq, we constructed saf file which considers TE subfamily as meta-feature and used featureCounts to assign mapped reads to the corresponding TE subfamily.

### Ultra-low-input micrococcal nuclease-based native ChIP (ULI-NChIP)

For ULI-NChIP-seq, about 5 × 10^4^ naïve or primed cells were used per reaction. The ULI-NChIP procedure was performed as previously described [103, 104]. Sequencing libraries were generated using AT seq library following manufacturer’s recommendations and index codes were added. The library quality was assessed on the Qubit 2.0 Fluorometer (Thermo Scientific) and Agilent Bioanalyzer 2100 system. At last, the library was sequenced on an Illumina HiSeq x platform and 150 bp paired-end reads were generated.

### Whole-exome sequencing

Paired-end DNA library was prepared according to manufacturer’s instructions (Agilent). Genomic DNAs (gDNA) from cell samples were sheared into 180–280 bp fragments by Covaris S220 sonicator. Ends of gDNA fragments were repaired and 3′ ends were adenylated. Both ends of gDNA fragments were ligated at the 3′ ends with paired-end adaptors (Illumina) with a single ‘T’ base overhang and purified using AMPure SPRI beads from Agencourt. The adaptor-modified gDNA fragments were enriched by six cycles of PCR using SureSelect Primer and SureSelect ILM Indexing PreCapture PCR Reverse Primer. The concentration and size distribution of the libraries were determined on an Agilent Bioanalyzer DNA 1000 chip. Whole-exome capture was carried out using SureSelect Mouse All Exon V1 Agilent 5190-4642. An amount of 0.5 μg prepared gDNA library was hybridized with capture library for 5 min at 95 °C followed by 24 h at 65 °C. The captured DNA–RNA hybrids were recovered using Dynabeads MyOne Streptavidin T1. Capture products were eluted from the beads and desalted using Qiagen MinElute PCR purification columns. The purified capture products were then amplified using the SureSelect ILM Indexing Post Capture Forward PCR Primer and PCR Primer Index (Agilent) for 12 cycles. After DNA quality assessment, captured DNA library was sequenced on Illumina Hiseq 2000 sequencing platform (Illumina) according to the manufacturer’s instructions for paired-end 150 bp reads (Novogene). Libraries were loaded onto paired-end flow cells at concentrations of 14–15 pM to generate cluster densities of 800,000–900,000 per mm^2^ using Illumina cBot and HiSeq paired-end cluster kit.

### Whole-exome sequencing data analysis

The analysis was performed according to the protocols specified for mouse cells [105]. Briefly, the adaptors of raw reads were trimmed by cutadapt, then bwa was used to map reads to mm10 genome. Picard was used to process the aligned reads and GATK to recalibrate the base quality scores. Copy number variant detection was used by software package CNVkit [106], which used both the targeted reads and the nonspecifically captured off-target reads to infer copy number evenly across the genome. We used segmentation algorithm hmm and set threshold of 500 bp and higher to define CNVs. To obtain the de novo CNVs from primed cells, we pooled all the naïve samples served as a reference and filtered out the CNVs shared among four (CBA × C57) or three (129 × C57) primed cell line clones. TE annotation file RepeatMasker (downloaded from UCSC) was used to obtain the number and type of TEs contained within CNVs.

### Statistical analysis

Data were analyzed by Student’s t test if not indicated. Overlap of upregulated genes in naïve and primed cells and 2C genes shown by Venn diagram was analyzed by Fisher’s exact test. Wald test was used for analysis of differentially expressed genes and TE locus. Wilcoxon test was used for analysis of differentially expressed TE locus derived from FPKM value and differentially enriched ChIP-seq signal on 2C genes. Significant differences were defined as *P< 0.05, **P< 0.01, or ***P< 0.001.

## Supplementary Information

**Additional file 1: Figure S1**. Pluripotency and developmental potential of naïve mESCs and primed mEpiSCs. **Figure S2**. Developmental potential of naïve mESCs and primed mEpiSCs *in vivo*. **Figure S3**. Comparison of naïve and primed pluripotent state *in vitro* and *in vivo* ICM and post-implantation epiblast counterpart. **Figure S4**. Comparison of naïve and primed pluripotent state by GO analysis related to DNA repair pathways. **Figure S5**. Differential telomere length dynamics in naïve and primed state cells at 129 × C57BL/6 genetic background. **Figure S6**. DNA recombination repair genes link to telomere maintenance in naïve mESCs. **Figure S7**. Methylation and histone modifications in naïve and primed ESCs. **Figure S8**. Histone modifications and Dnmt3b enrichment at naïve and primed pluripotent genes and specific TEs. **Figure S9**. Kap1-mediated H3K9me3 regulates *L1Md_T* and *IAPEz-int* transcription in naïve and primed PSCs. **Figure S10**. Uncropped scans of Western blot with molecular weight markers.

**Additional file 2: Table S1**. Differential gene transcriptome at passage 5.

**Additional file 3: Table S2**. Differential gene transcriptome at passage 15.

**Additional file 4: Table S3**. Differential TE transcriptome at passage 5.

**Additional file 5: Table S4**. Differential TE transcriptome at passage 15.

**Additional file 6: Table S5**. Summary of CNVs containing TEs in naïve and primed PSCs in two different genetic background cell lines.

**Additional file 7: Table S6**. Key resource table.

**Additional file 8. ** Review history.

## Data Availability

RNA-seq data and ChIP-seq data are available from Gene Expression Omnibus under accession number GEO: GSE140665 [107] and GSE154487 [108]. The accession number for Exome-seq data is NCBI Sequence Read Archive: PRJNA725383 [109]. All public data used in this study have been listed and well referenced in Additional file 7: Table S6, including in vivo embryos RNA-seq data: GSE98150 [104], RNA-seq data from *Setdb1* KO mESC: PRJNA544540 [70], *G9a* KO mESC: GSE49669 [71], *Dnmt3b* KO mESC: GSE72855 [72] and *Kap1* KD mESC: GSE95720 [76], and H3K9me3 and Kap1 ChIP-seq data: GSE94323 [75]. All cell lines used in this study have been authenticated and are available upon request.
