# Supplementary material for: Elevated retrotransposon activity and genomic instability in primed pluripotent stem cells
Source: Genome Biol. 2021 Jul 9;22:201. doi: 10.1186/s13059-021-02417-9 (PMC8268579; doi:10.1186/s13059-021-02417-9)
Supplement: Supplementary file 1 — Additional file 1: Figure S1. Pluripotency and developmental potential of naïve mESCs and primed mEpiSCs. Figure S2. Developmental potential of naïve mESCs and primed mEpiSCs in vivo. Figure S3. Comparison of naïve and primed pluripotent state in vitro and in vivo ICM and post-implantation epiblast counterpart. Figure S4. Comparison of naïve and primed pluripotent state by GO analysis related to DNA repair pathways. Figure S5. Differential telomere length dynamics in naïve and primed state cells at 129 × C57BL/6 genetic background. Figure S6. DNA recombination repair genes link to telomere maintenance in naïve mESCs. Figure S7. Methylation and histone modifications in naïve and primed ESCs. Figure S8. Histone modifications and Dnmt3b enrichment at naïve and primed pluripotent genes and specific TEs. Figure S9. Kap1-mediated H3K9me3 regulates L1Md_T and IAPEz-int transcription in naïve and primed PSCs. Figure S10. Uncropped scans of Western blot with molecular weight markers. [file 13059_2021_2417_MOESM1_ESM.docx]

**SUPPLEMENTARY INFORMATION**

**Additional file 1**

Additional file 1: Figure S1. Pluripotency and developmental potential of naïve mESCs and primed mEpiSCs. Figure S2. Developmental potential of naïve mESCs and primed mEpiSCs *in vivo*. Figure S3. Comparison of naïve and primed pluripotent state *in vitro* and *in vivo* ICM and post-implantation epiblast counterpart. Figure S4. Comparison of naïve and primed pluripotent state by GO analysis related to DNA repair pathways. Figure S5. Differential telomere length dynamics in naïve and primed state cells at 129×C57BL/6 genetic background. Figure S6. DNA recombination repair genes link to telomere maintenance in naïve mESCs. Figure S7. Methylation and histone modifications in naïve and primed ESCs. Figure S8. Histone modifications and Dnmt3b enrichment at naïve and primed pluripotent genes and specific TEs. Figure S9. Kap1-mediated H3K9me3 regulates *L1Md_T* and *IAPEz-int* transcription in naïve and primed PSCs. Figure S10. Uncropped scans of Western blot with molecular weight markers.

**Additional file 2: Table S1.**

Differential gene transcriptome at passage 5.

**Additional file 3: Table S2.**

Differential gene transcriptome at passage 15.

**Additional file 4: Table S3.**

Differential TE transcriptome at passage 5.

**Additional file 5: Table S4.**

Differential TE transcriptome at passage 15.

**Additional file 6: Table S5.**

Summary of CNVs containing TEs in naïve and primed PSCs in two different genetic background cell lines.

**Additional file 7: Table S6.**

Key resource table.

**Additional file 8: Review history.**

**
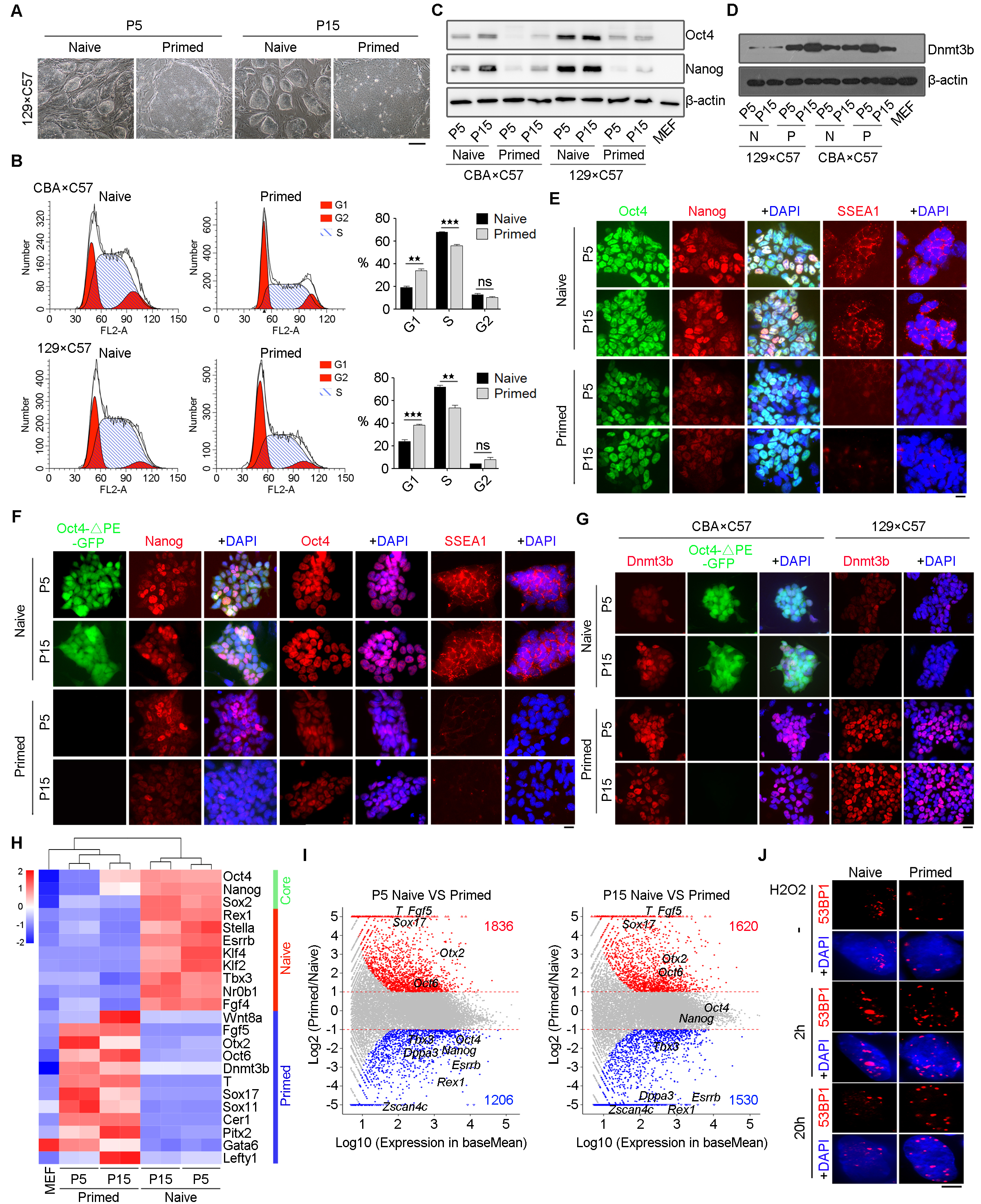
**

**Figure S1. Pluripotency and developmental potential of naïve mESCs and primed mEpiSCs. Related to Figure 1**

(A) Representative morphology of 129×C57 naïve mESCs and primed mEpiSCs on P5 and P15 under bright field with phase contrast optics. Scale bar = 100 µm.

(B) Cell cycle analysis of P5 naïve and primed cells by flow cytometry. Error bars indicate mean ± SEM (n = 3). **P < 0.01 and ***P < 0.001.

(C) Nanog and Oct4 protein levels by western blot analysis.

(D) Dnmt3b protein levels by western blot analysis.

(E and F) Immunofluorescence of pluripotent markers Oct4, Nanog, and SSEA1 in 129×C57 (E) and CBA×C57 (F) cell lines. Scale bar = 10 µm.

(G) Immunofluorescence microscopy of primed state marker Dnmt3b in two cell lines. Scale bar = 10 µm.

(H) Heatmap highlighting expression profile and patterns of marker genes for CBA×C57 naïve and primed state cells and MEF cells served as controls.

(I) Scatter-plots comparing genome-wide transcription profile between CBA×C57 naïve and primed state cells at P5 and P15. Parallel diagonal lines indicate two-fold threshold in expression difference.

(J) 129×C57 primed cells by exposure to 5 mM H2O2 for 2 h exhibit more 53BP1 foci after 20 h recovery compared with naïve cells by immunofluorescence. Scale bar = 5 μm.

**
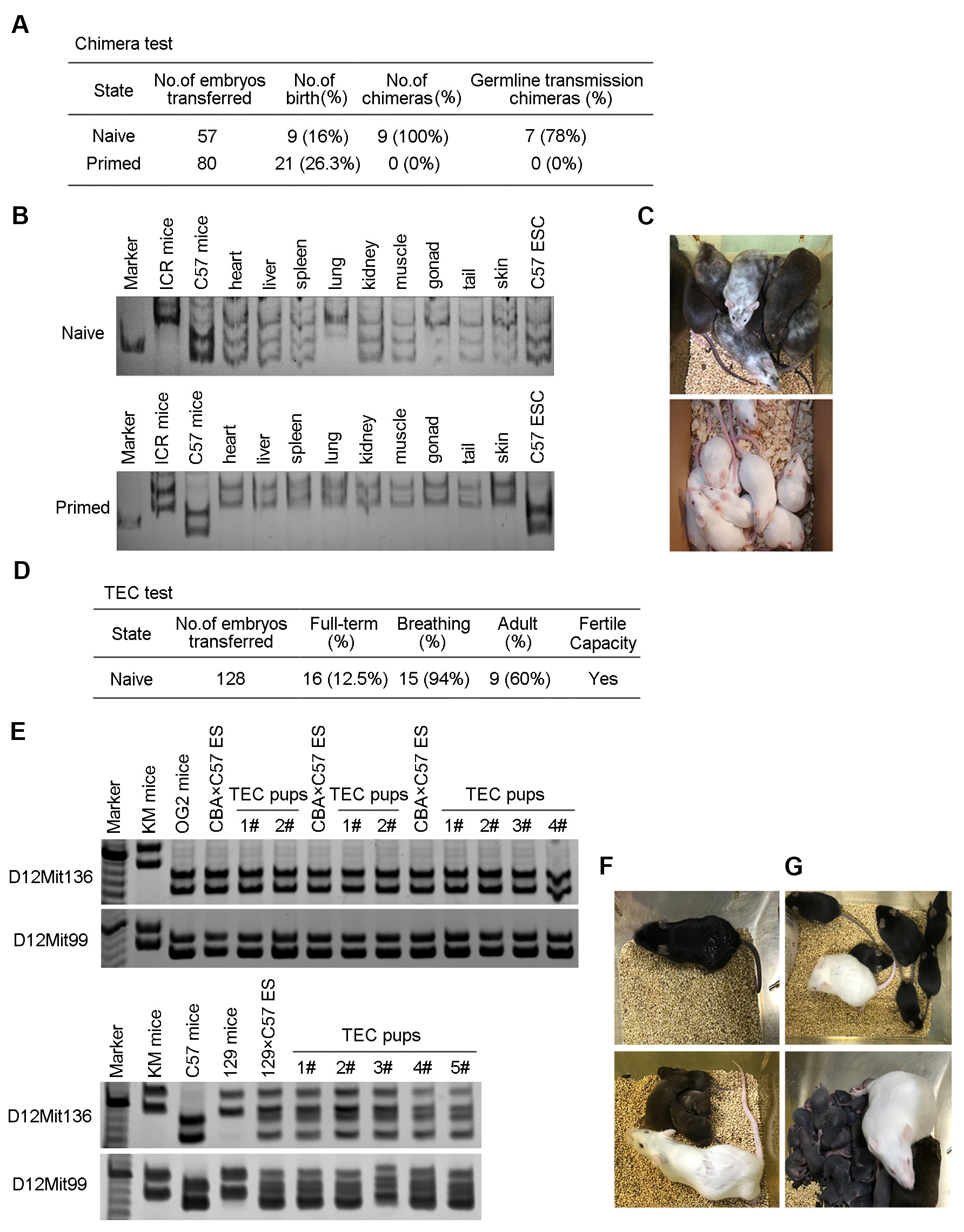
**

**Figure S2. Developmental potential of naïve mESCs and primed mEpiSCs *in vivo*.** **Related to Figure 1**

(A) Statistics of 4-8 cell injection by naïve and primed cells. Naïve mESCs at P10-P14 were used to generate chimeras with germline transmission capacity by 4-8 cell injection. Total 57 embryos were transferred into 2 surrogate mothers. P12+4, P12+13 and P9+13 primed mEpiSCs were used for chimera test. Total 80 embryos were transferred into 4 surrogate mothers.

(B) Microsatellite genotyping analysis showing contribution of naïve and primed cells to various tissues of the chimeras. 4-8 cell embryos from ICR mice were used as recipients. Arrows indicate donor cells, C57 mice and contributions in the chimeras.

(C) Upper photo represents chimeras generated from naïve mESCs and the bottom from primed mEpiSCs at P14-16.

(D) Summary of TEC experiment by naïve mESCs. naïve mESCs at P5-P10. Total 128 embryos were transferred into 4 surrogate mothers.

(E) Microsatellite genotyping analysis showing contribution of naïve mESCs to TEC pups. Upper image represents TEC pups from naïve CBA×C57 mESCs, bottom image represents TEC pups from 129×C57 naïve mESCs.

(F) Complete mESC-derived pups by TEC. Upper photo represents TEC mouse generated from CBA×C57 naïve mESCs and the bottom from 129×C57 mESCs.

(G) Offspring from TEC mice by mating with ICR mice. Upper photo represents offspring from TEC mouse derived from CBA×C57 mESCs and the bottom from TEC mouse derived from 129×C57 mESCs.


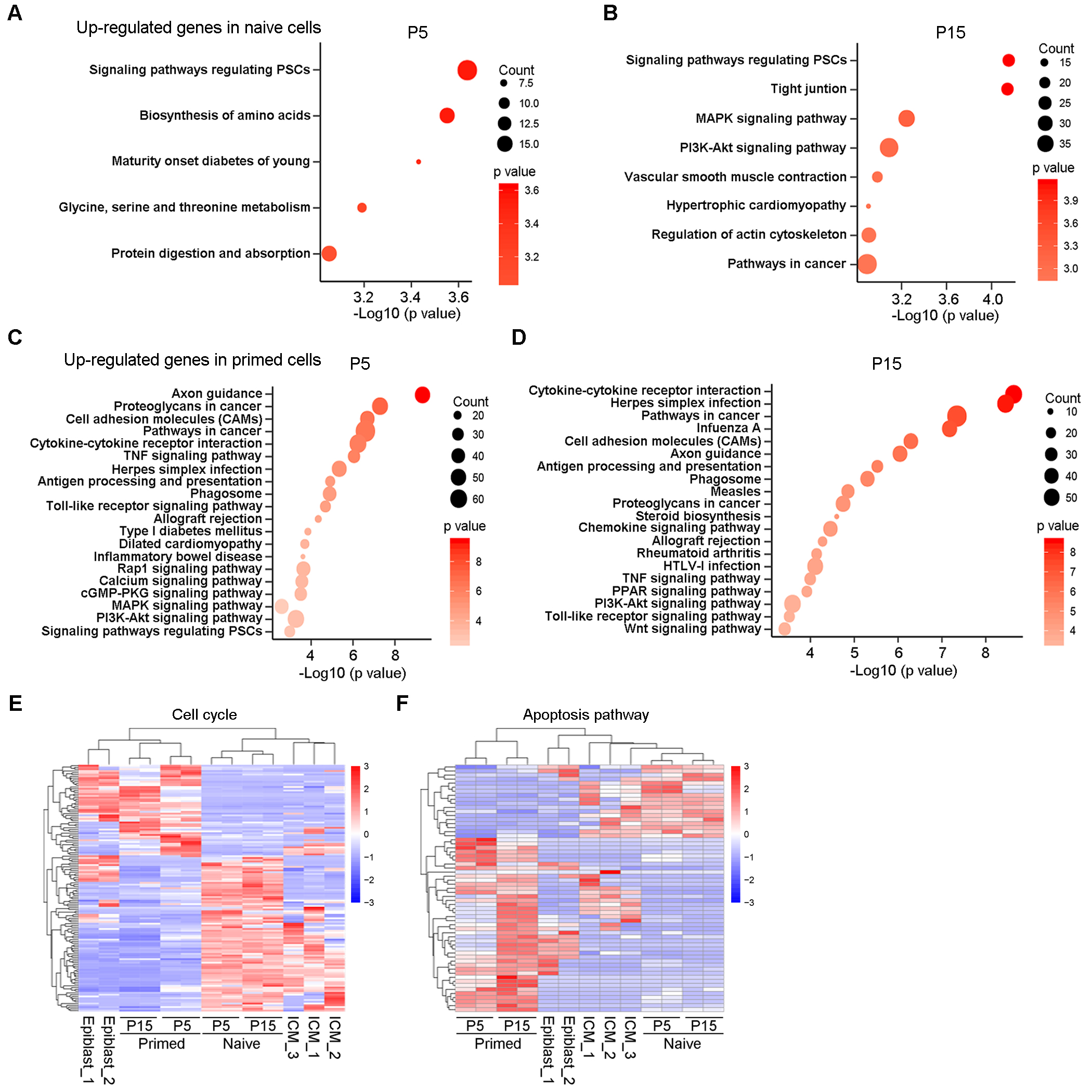


**Figure S3. Comparison of naïve and primed pluripotent state *in vitro* and *in vivo* ICM and post-implantation epiblast counterpart.** **Related to Figure 1**

(A and B) KEGG analysis of up-regulated genes in naïve cells compared with the primed cells at P5 (A) and P15 (B).

(C and D) KEGG analysis of up-regulated genes in primed cells compared with the naïve cells at P5 (C) and P15 (D).

(E and F) Heatmap showing cell cycle (E) and apoptosis (F) pathway in naïve and primed cells compared with their *in vivo* counterparts ICM and post-implantation epiblast (*in vivo* data from [104]).


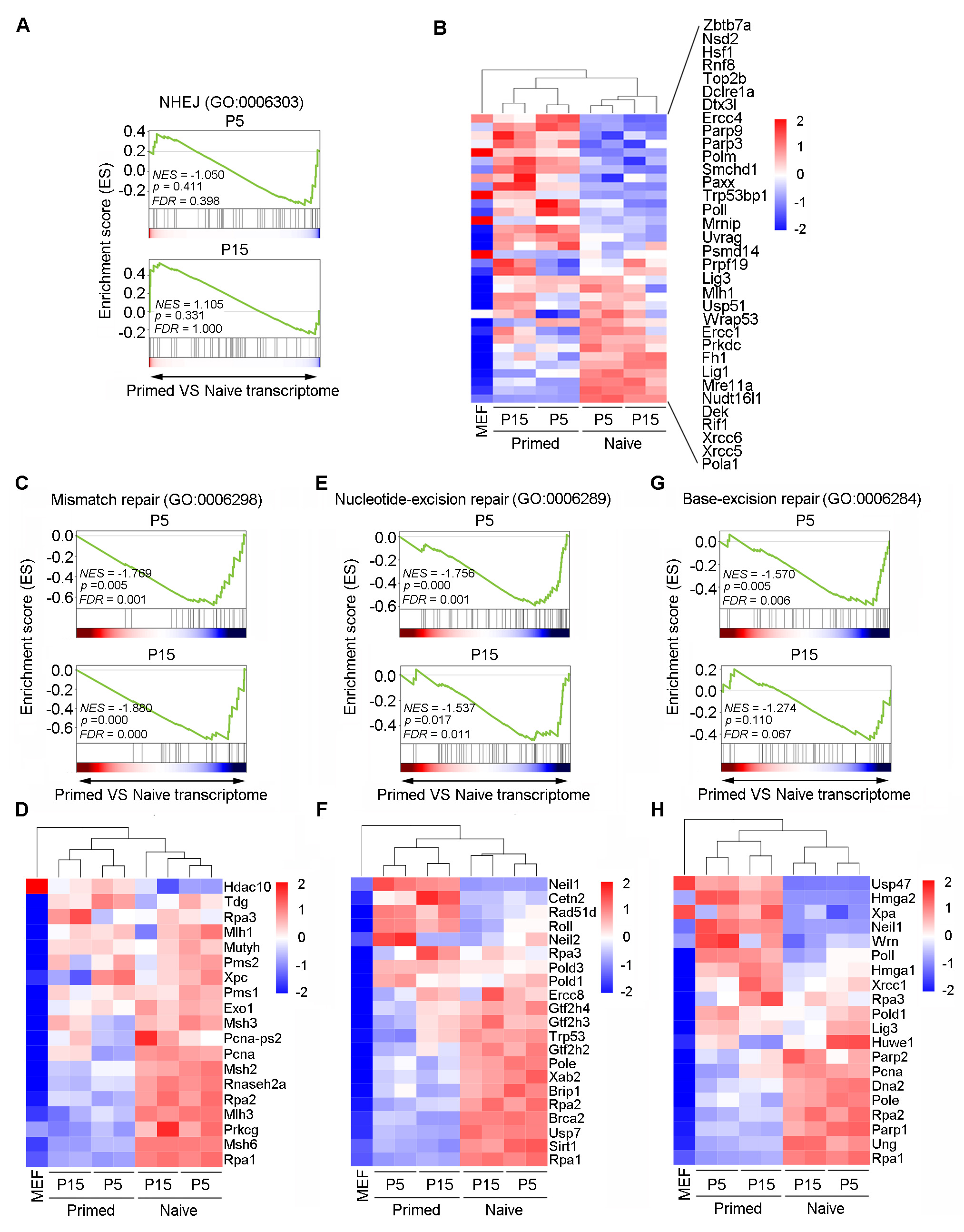


**Figure S4. Comparison of naïve and primed pluripotent state by GO analysis related to DNA repair pathways.** **Related to Figure 1**

(A and B) GSEA analysis and heatmap showing DNA non-homologous end joining (NHEJ) pathway in MEF cells, and naïve and primed cells at P5 and P15.

(C and D) GSEA analysis and heatmap showing DNA mismatch repair pathway in MEF cells, and naïve and primed cells at P5 and P15.

(E and F) GSEA analysis and heatmap showing DNA nucleotide-excision repair pathway in MEF cells, and naïve and primed cells.

(G and H) GSEA analysis and heatmap showing DNA base-excision repair pathway in MEF cells, and naïve and primed cells.

**
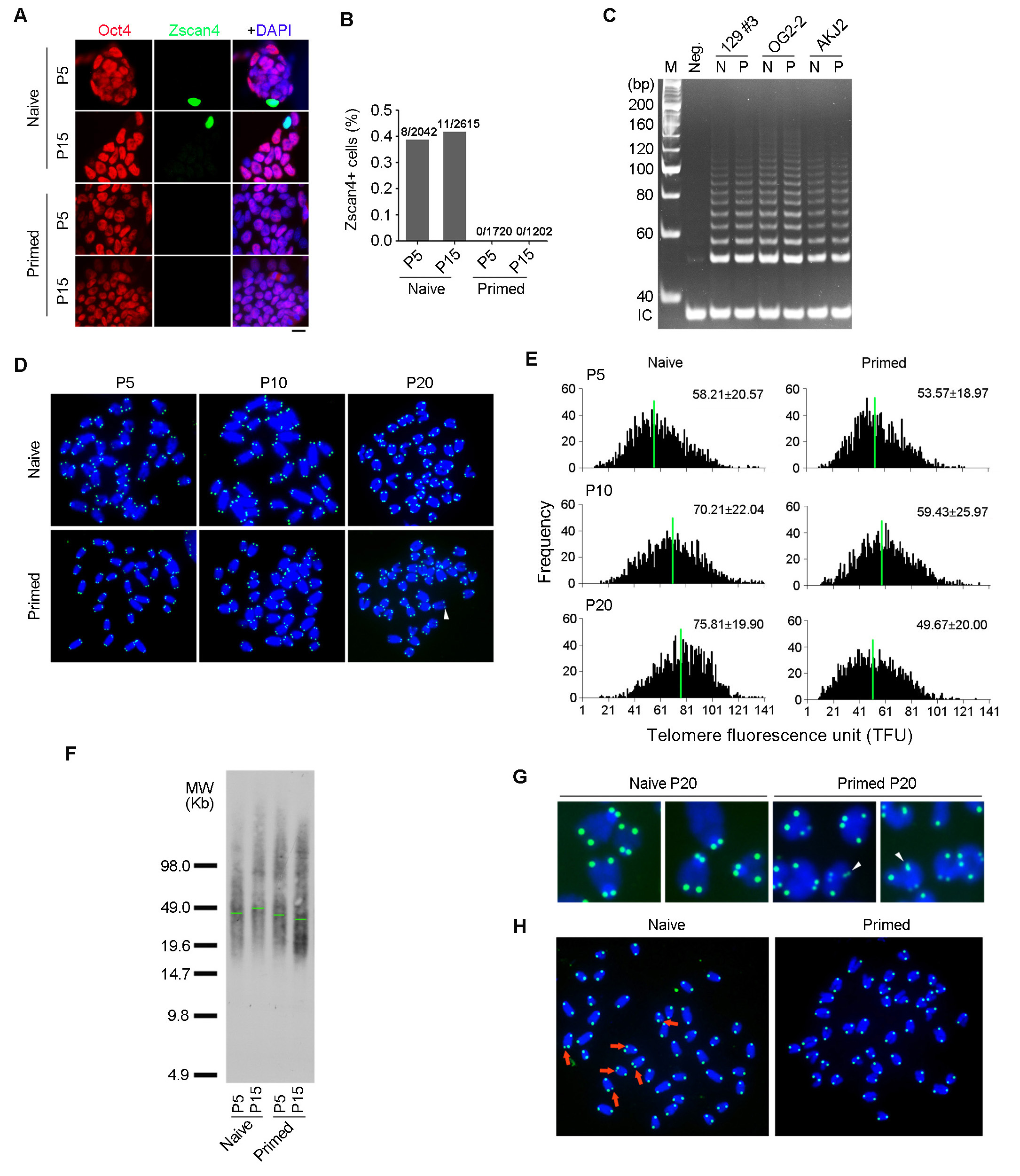
**

**Figure S5. Differential telomere length dynamics in naïve and primed state cells at 129×C57BL/6 genetic background. Related to Figure 2 and Figure 3**

(A) Immunofluorescence of Zscan4 in 129×C57 naïve and primed cells cultured at P5 and P15. Scale bar = 10 µm.

(B) Proportion of Zscan4^+^ cells by immunofluorescence image. Counted number is indicated at the top of the bar.

(C) Telomerase activity by TRAP assay of three different cell lines at naïve and primed state.

(D) Representative images displaying telomere FISH at P5, P10 and P20 129×C57 naïve and primed PSCs. Blue, chromosomes stained with DAPI; green dots, telomeres. The white arrows indicate fragile telomeres.

(E) Histogram shows distribution of relative telomere length displayed as TFU by Q-FISH analysis. Green line indicates medium telomere length. Mean ± s.d. of telomere length is shown above each panel.

(F) Telomere length distribution shown as TRF by Southern blot analysis of 129×C57 naïve and primed PSCs. Green lines indicate average telomere length.

(G) Enlarged telomere Q-FISH images, showing telomere fragility (white arrowhead) in129×C57 primed PSCs at P20, but not in naïve PSCs. Telomeres were labeled with telomere PNA probes (green), and chromosomes labeled with DAPI (blue).

(H) Representative micrographs showing telomere sister chromatid exchange (T-SCE, red arrows) by chromosome orientation FISH analysis of 129×C57 cell line.


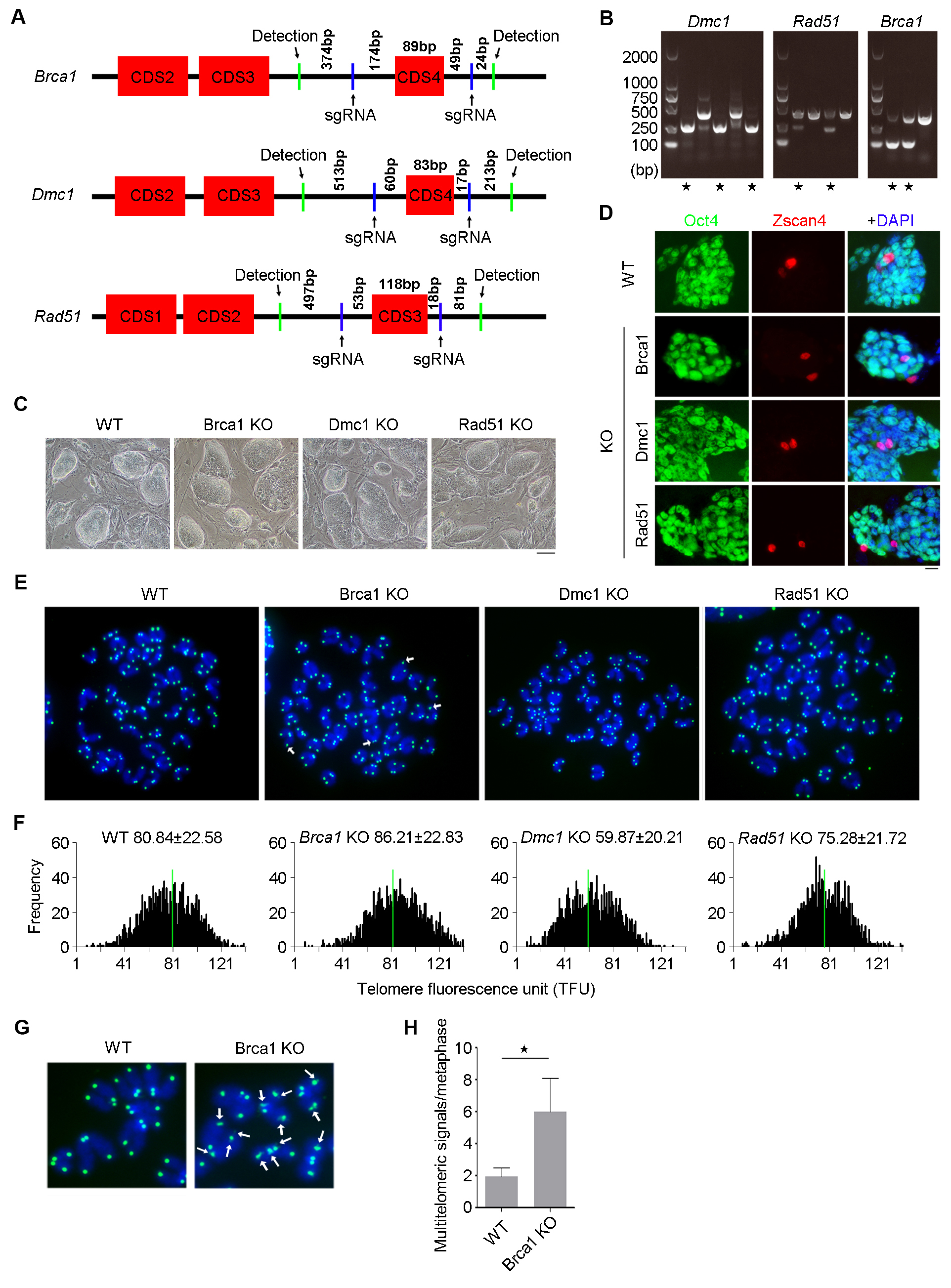


**Figure S6.** **DNA recombination repair genes link to telomere maintenance in naïve mESCs.** **Related to Figure 3**

(A) Schematic of Cas9/sgRNA-targeting sites of *Brca1*, *Dmc1* or *Rad51*. Sites of cleavage by Cas9 are indicated by blue lines. Detection forward and reverse primers are shown as green lines.

(B) Genotyping of *Brca1*, *Dmc1* or *Rad51* knockout (KO) clones generated by CRISPR/Cas9.

(C) Colony morphology of WT and *Brca1*, *Dmc1* and *Rad51* KO mESCs at P15+8. Scale bar = 100 μm.

(D) *Brca1*, *Dmc1* or *Rad51* KO does not alter expression of Oct4 and Zscan4 by fluorescence microscopy. Scale bar = 10 μm.

(E) Representative images displaying telomere FISH of WT, *Brca1*, *Dmc1* and *Rad51* KO mESCs at 129×C57 background at P15+8. Blue, chromosomes stained with DAPI; green dots, telomeres. The white arrows indicate fragile telomeres.

(F) Histogram shows distribution of relative telomere length displayed as TFU by Q-FISH analysis. Green line indicates medium telomere length. Mean ± s.d. of telomere length is shown above each panel.

(G) Telomere Q-FISH images at large scale, showing telomere fragility (white arrowhead) in *Brca1* KO mESCs. Telomeres were labeled with telomere PNA probes (green), and chromosomes labeled with DAPI (blue).

(H) Frequency of telomere fragility per chromosome in G.

**
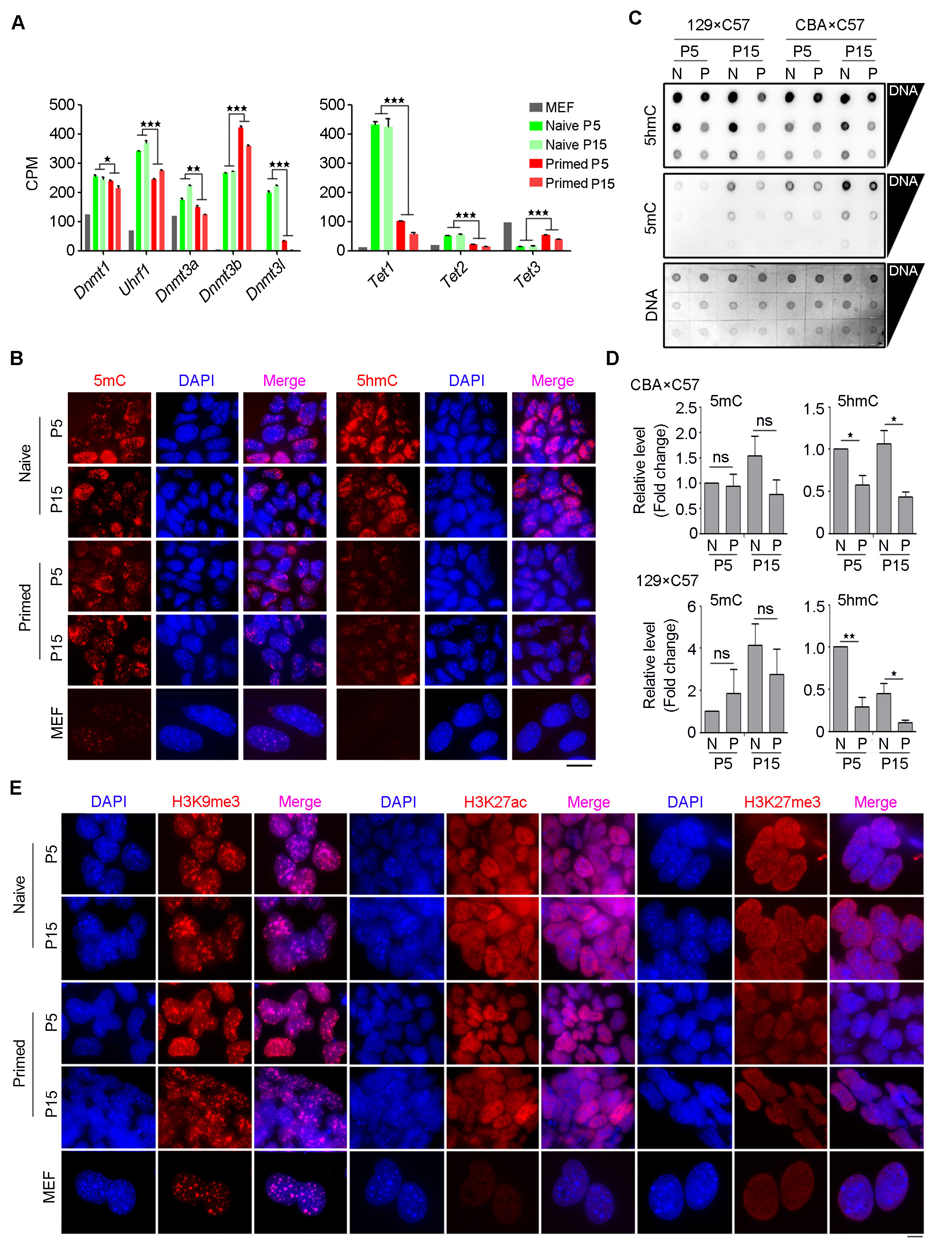
**

**Figure S7. Methylation and histone modifications in naïve and primed ESCs. Related to Figures 4 and 5**

(A) Expression levels (CPMs) of multiple genes involved in DNA demethylation.

(B) Immunofluorescence of 5mC and 5hmC in naïve and primed cells at P5 and P15 and MEF cells. Scale bar = 10 µm.

(C) Dot blot analysis showing global DNA methylation and hydroxymethylation levels in two cell lines at P5 and P15.

(D) Quantification of relative 5mC and 5hmC intensity using Image J software. *p < 0.05, **p < 0.01.

(E) Immunofluorescence of relative histone epigenetic modifications in naïve and primed cells at P5 and P15 and MEF cells. Scale bar = 10 µm.

**
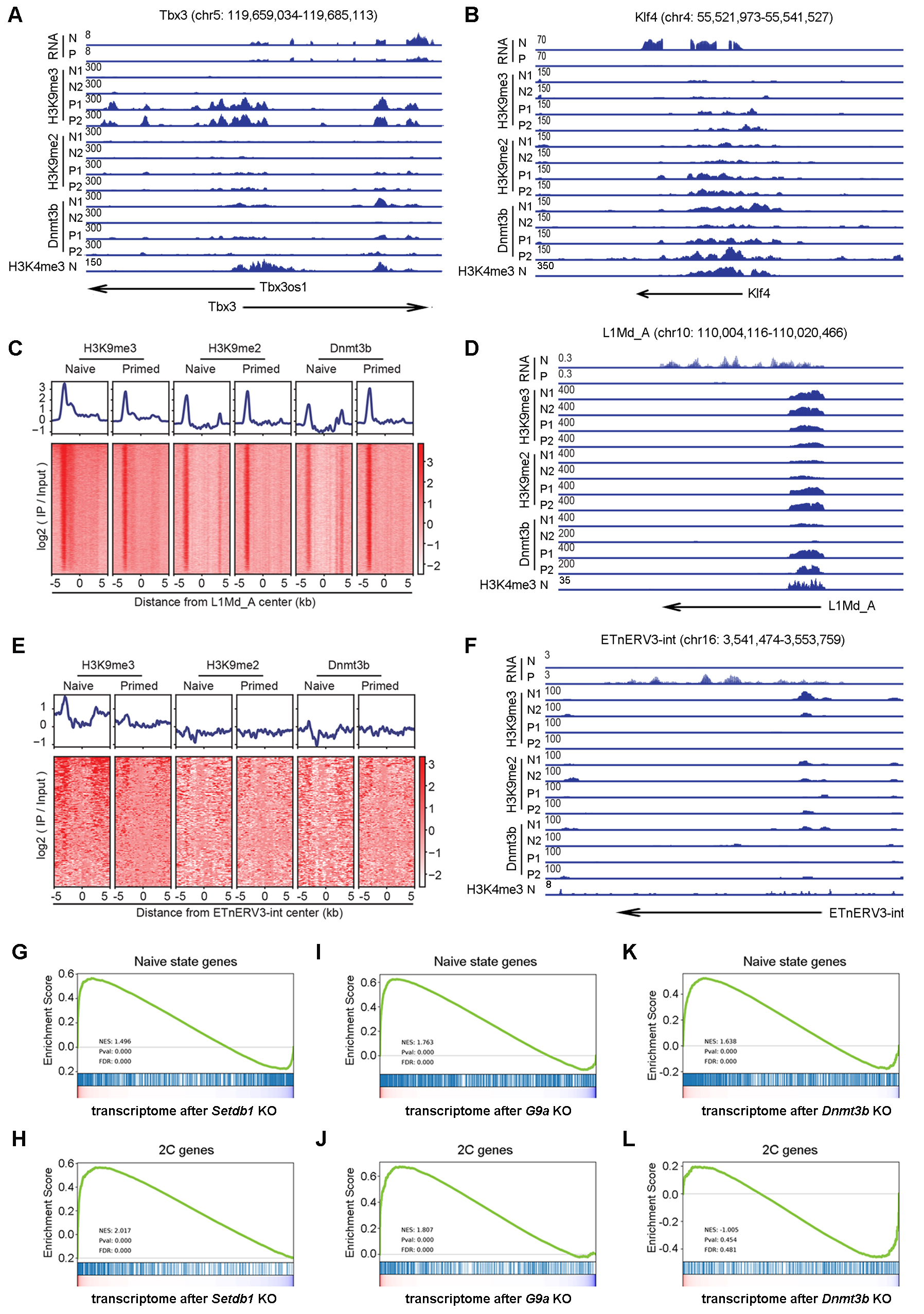
**

**Figure S8. Histone modifications and Dnmt3b enrichment at naïve and primed pluripotent genes and specific TEs. Related to Figure 5**

(A, B, D, F) Density plot of RNA-seq and ChIP-seq signal of *Tbx3*, *Klf4*, *L1Md_A* and *ETnERV3-int* loci. Arrow indicates the direction of transcription. H3K4me3 were used to indicate the promoters of genes. RNA-seq signals are normalized by CPM, ChIP-seq signals are normalized by RPKM.

(C, E) Plot of H3K9me3, H3K9me2 and Dnmt3b binding profile and heatmap at all *L1Md_A* and *ETnERV3* loci in naïve and primed PSCs. The ChIP-seq signals are calculated as log2 ratio of normalized reads relative to the input.

(G, H) Gene set enrichment analysis (GSEA) indicating that upregulated naïve state genes and 2C genes after *Setdb1* knockout (KO) in naïve mESCs (data from [70]).

(I, J) Gene set enrichment analysis (GSEA) indicating that upregulated naïve state genes and 2C genes after *G9a* KO in naïve mESCs (data from [71]).

(K, L) Gene set enrichment analysis (GSEA) indicating that upregulated naïve state genes and unaffected 2C genes after *Dnmt3b* KO in naïve mESCs (data from [72]).

**
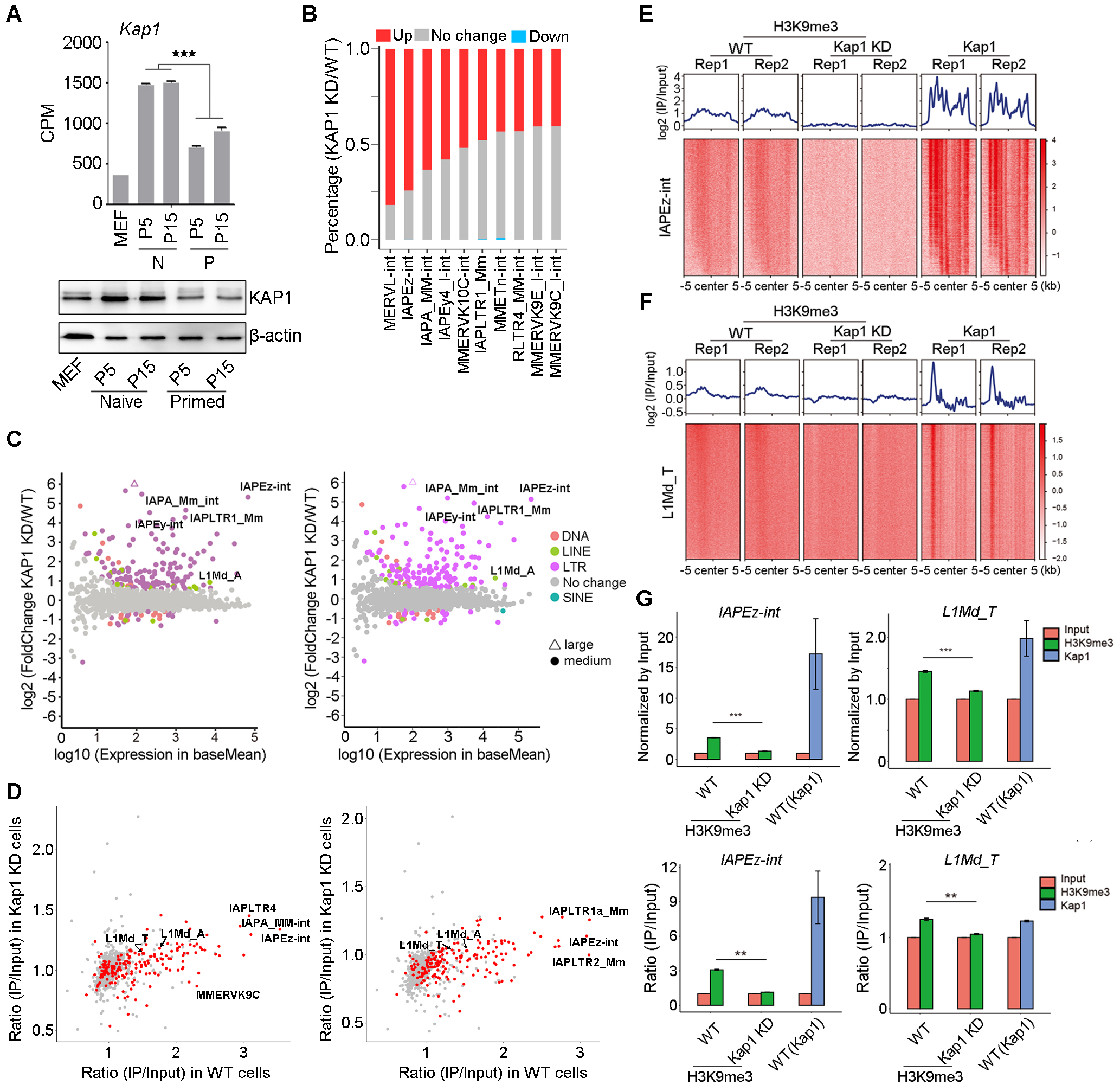
Figure S9. Kap1-mediated H3K9me3 regulates *L1Md_T* and *IAPEz-int* transcription in naïve and primed PSCs. Related to Figure 5**

(A) Expression level (CPM) and protein level of Kap1 in naïve and primed cells at P5 and P15 and MEFs.

(B) TE signatures after *Kap1* knockdown (KD) in mESCs showing that most TEs are up-regulated, and *MERVL* and *IAPEz-int* are the most significantly up-regulated.

(C) Scatter plot figure showing change of TEs after *Kap1* KD in mESCs by uniquely mapped reads (left panel) and multi-mapped reads (right panel). The count of TE subfamilies is the sum of count of corresponding loci.

(D) Scatter plot for ChIP-signal on TE subfamilies by uniquely mapped reads (left panel) and multi-mapped reads (right panel). Ratio of cpm of IP/Input was used. Significance was calculated by Student's t-test. p < 0.05 was considered significant and label as red.

(E) H3K9me3 and Kap1 binding profile plot and enrichment heatmap at *IAPEz-int* site in WT and *Kap1* KD mESCs. The ChIP-seq signals were calculated as the ratio of normalized reads relative to input.

(F) H3K9me3 and Kap1 binding profile plot and enrichment heatmap at *L1Md_T* site in WT and *Kap1* KD mESCs. The ChIP-seq signals were calculated as the ratio of normalized reads relative to input.

(G) Enrichment of H3K9me3 and Kap1 by ChIP-seq on *IAPEz-int* and *L1Md_T* by uniquely mapped reads (upper panel) and multi-mapped reads (bottom panel). Ratio of cpm of IP/Input was used. Significance was calculated by Student's t-test. **p < 0.01 and ***p < 0.001.

Results from Figures B-G are analyzed from published data [75,76].


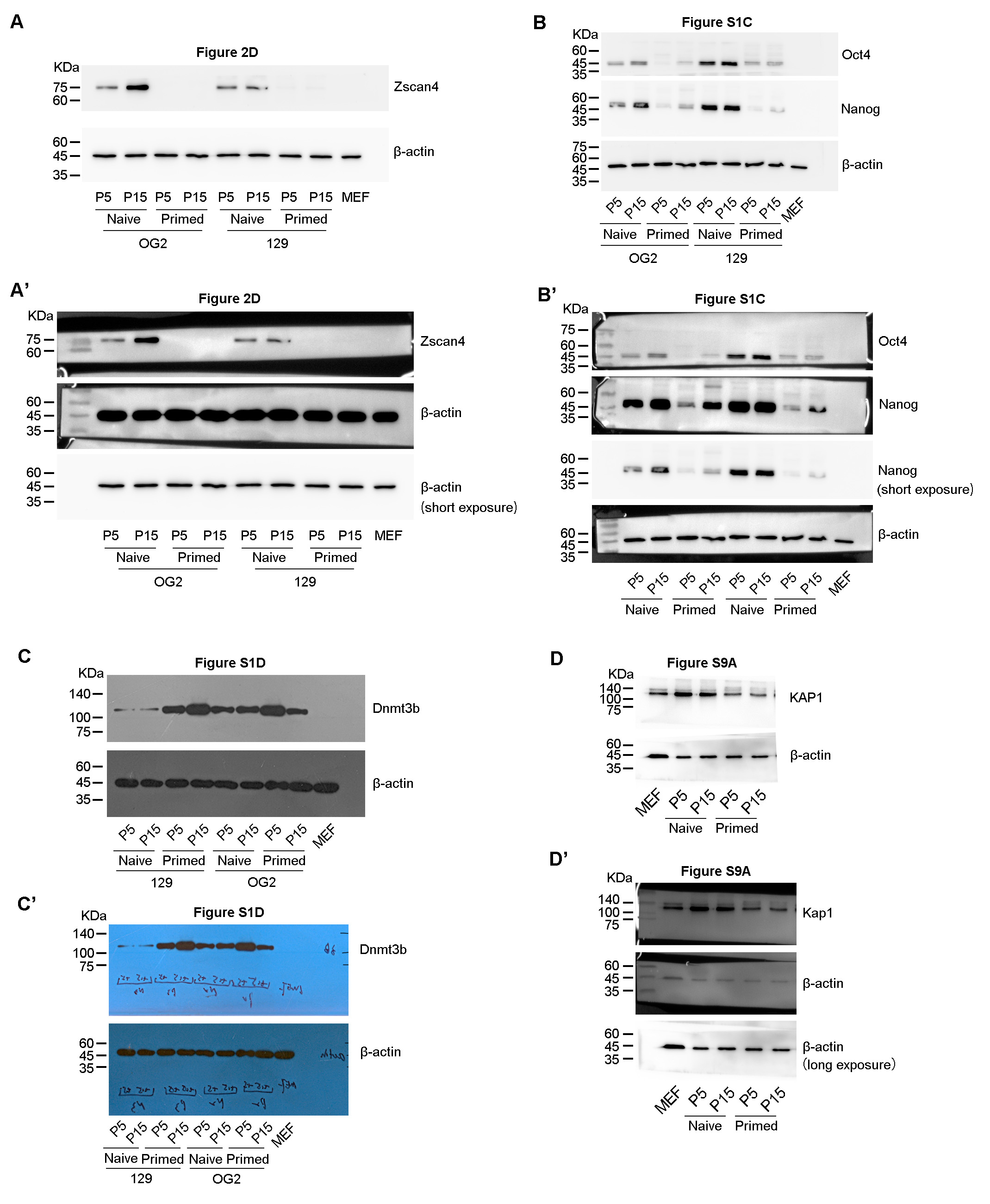


**Figure S10. Uncropped scans of Western blot with molecular weight markers.**

(A and A’) The uncropped Western blot for Figure 2D comparing the Zscan4 protein level in naïve and primed PSCs from two genetic background and MEFs served as negative control. β-actin served as loading control. β-actin band with short exposure time was used in Figure 2D. (A’) was the same gel as the image of (A) but with protein markers under bright field, and the black background was from exposure machine panel.

(B) The uncropped Western blot for Figure S1C comparing the Oct4 and Nanog protein levels in naïve and primed PSCs from two genetic background and MEFs served as negative control. β-actin served as loading control. Nanog with short exposure band was used in Figure S1C. (B’) was the same gel as the image of (B) but with protein markers under bright field, and the black background was from exposure machine panel.

(C) The uncropped Western blot for Figure S1D comparing the Dnmt3b protein level in naïve and primed PSCs from two genetic background and MEFs. β-actin served as loading control. (C’) was the original X-ray film of (C), but the markers could not appear on the X-ray film, so the markers appeared on the transferred PVDF membrane were copied on the right hand side.

(D) The uncropped Western blot for Figure S9A comparing the Kap1 protein level in CBA×C57 naïve and primed PSCs and MEFs. β-actin served as loading control. β-actin band by long exposure time was used in Figure S9A. (D’) was the same gel as the image of (D) but with protein markers under bright field, and the black background was from exposure machine panel.
