# Supplementary material for: Elevated retrotransposon activity and genomic instability in primed pluripotent stem cells
Source: Genome Biol. 2021 Jul 9;22:201. doi: 10.1186/s13059-021-02417-9 (PMC8268579; doi:10.1186/s13059-021-02417-9)
Supplement: Supplementary file 8 — Additional file 8. Review history. [file 13059_2021_2417_MOESM8_ESM.docx]

Review History

**First round of review**

**Reviewer 1**

**Are you able to assess all statistics in the manuscript, including the appropriateness of statistical tests used? Yes, and I have assessed the statistics in my report.**

**Comments to author:**

This is impressive work, covering a wide range of techniques for discovering the molecular determinants of naive / primed states in murine cells. This topic has been covered by several previous posts, and therefore some parts are lacking in novelty, but many sections are new. Overall, this work highlights a critical point in initiated PSC, including that lower telomere maintenance results in telomere fragility, and that there is increased genomic instability related to retrotransposon activity. Overall, the scope of this work may make it a benchmark in the field of naive / primed pluripotency in rodents.

Major

1 / A major limitation of the study is the lack of indication of repeatability. For example, in Figure 3 E-G-H: how many times was the experiment repeated, how many experimental time points are shown in the figures? For bar charts, it is now common to overlay bar charts with bar charts. This should be preferred, as it explicitly states how many data points were used to build the graph. Similar note for Figure 6: The experiments in this figure are essential for the conclusion of the manuscript, however, there is evidence that each experiment was performed only once. Such a decisive experiment must be repeated at least three times.

2 / In FIG. 6E, the gains> = 5 are the most frequent events. It is understood that this is due to TE, but the authors should explain in more depth how this occurs. What is the percentage of CNV with at least one TE, and up to how much? Where available, do CNV boundaries provide any indication of the mechanism underlying the induction of CNV by TE? Discuss this part in more detail. Also, how do variations of CNV in primed cells compare to a more differentiated cell type? Discuss.

3 / In the abstract, the authors suggest that "telomere fragility and retrotransposon-induced genomic instability, associated with decreased DNA recombination repair, may be related to the compromised developmental potency of primed PSCs, noticeably distinguishable from naive PSCs ". This should be discussed in the discussion section. It should be noted that genetic instability is only one of the many hypotheses that could explain the low developmental power of initiated PSCs. Other hypotheses include the differential cell cycle with ICM, poor survival in the ICM setting, or decreased differentiation properties.

**Reviewer 2**

**Are you able to assess all statistics in the manuscript, including the appropriateness of statistical tests used? No, I do not feel adequately qualified to assess the statistics.**

**Comments to author:**

In "Elevated retrotransposon activity and genomic instability in primed pluripotent stem cells", Fu et all aim at elucidating mechanisms that could be involved in the lower developmental potency of primed pluripotent stem cells, when compared to naïve embryonic stem cells.

While the subject is an important one, I feel that this manuscript is too speculative and lacks functional evidence for the authors conclusions (which are many, in their Discussion Section). In addition, by combining telomere data with retrotransposon analysis, with the lack of any mechanistical reasoning to do so, the manuscript becomes unnecessarily long and descriptive. A few more specific points bellow:

1)	Figure 1 doesn't add anything novel to the field. It is simply a description of gene expression profiles in naïve vs primed, which has been described many times before

2)	The authors argue (from GO analysis) that recombination and DNA damage pathways are reduced in the primed state, when compared to the naïve state. The authors reach this conclusion because RNASeq analysis show lower expression of HR, MMR and NER genes. However, the expression levels of these genes alone is far from being sufficient to reach such a conclusion. The authors should challenge both cell types to IR, ROS and UV light and quantify rates of DNA repair and cell survival.

3)	Authors argue a possible decrease in the OXPHOS/Glycolytic pathway in the transition from naïve to primed based on RNAseq. At the very minimal a Seahorse analysis should be performed to validate this claim.

4)	Expression of TERT and TERC are similar (Figure 3), and telomerase activity assays (unfortunately performed by PCR) reflect that. On the same figure, authors claim telomere elongation in naïve and not in primed cells. The difference however seems to be minimal and is difficult to analyze as we only see relative data (Figure 3C) and not telomere length by itself. The authors should show telomere length assays by TRF or STELLA. Arguably the Q-FISH analysis (3D/E) serves this purpose, but the elongation rates between that and 3C are discordant, and the standard deviation pretty significant.

5)	On page 19, line 25, the authors write: "Regardless of the mechanisms, this, along with the declined DNA recombination repair capacity […]". The fact is that one cannot disregard mechanism when making claims like this. Also, the authors don't show declined DNA repair capacity in any of their figures. Only expression data. Those are not the same. Too speculative.

6)	Page 20, line 50: The authors claim that "Reduced telomere recombination activity corroborates robust telomere lengthening in naïve PSCs". Again, functional data is lacking to support this. Manuscript has many associations, but a lack functional assays to support most of their claims.

7)	Page 23, line 36: Authors claim "These data suggested that diverse epigenetic modifications orchestrate in reducing naïve pluripotency gene expression and suppressing specific 2C genes in primed cells." This is too vague; no validation, just speculation.

8)	An important conclusion of the authors is that high Line1 and Alu activity, together with reduced telomere length in primed cells reduce the ability of primed ESCs to contribute to chimera. Again, no functional data to directly test this hypothesis is presented. If the telomeres get shorter and there is increased retrotransposition of Alu/L1 in primed cells with serial passage, then the chimera-forming ability of these cells at early passages should be similar to naïve cells, and decrease only after serial passaging. Authors should compare the chimera-forming ability of early (P5) versus late (P>30) primed ES cells and confirm this claim.

**Reviewer 3**

**Are you able to assess all statistics in the manuscript, including the appropriateness of statistical tests used? No, I do not feel adequately qualified to assess the statistics.**

**Comments to author:**

This work is a remarkable resource, consolidated by deeply analyses and many functional experiments, especially regarding telomeres. If the authors had added peri-centromic to telomeric regions and transposons, they would have truly performed a thorough analysis of DNA repeats, which would have given to their study a more definitive character ("mouse genomic repeat behaviors during naive to primed embryonic stem cells transition.") The part on TEs is not fundamentally novel, and some statements should be toned down, especially some of their conclusion about genomic stability.

Major comments:

1. Fig. 3. In addition to loss-of-function experiments, is gain of function sufficient to change the telomere's phenotype?

2. Fig. S7b. How do the authors reconcile their observations and previous reports indicating that naive mESC display decreased levels of DNA methylation and H3K27me3? This should be strongly discussed.

3. Fig. 5 and page 23 line 33. The authors did not need to "infer" that Dux4 might regulate 2-cell stage genes: that had already been demonstrated and published.

4. Fig. S9. The authors re-analyzed papers published years ago, when sequencing technology was still sub-optimal for proper mapping of retro-transposons. Moreover, the authors should also add up all reads from ChIP-seq and RNA-seq (including multimap) over each subfamily to make sure they do not just lose them when mapping uniquely. The authors may want also to re-analyze GSE95720, which represents a more recent dataset of KAP1 KD obtained using longer sequencing reads.

5. Fig. 6. How do the authors discriminate events which might appear randomly in individual cells from a bulk population? Isolation and sequencing of multiple clones should be performed. Are the observed retro-transposable elements enrichment difference between naive and primed ESC significant? Are there de novo insertions specific to primed versus naïve ESC? We are really not sure that these analysis support the claim of the authors.

Minor comments:

1. Some figures would deserve higher resolution.

2. Page 16 line 6: The authors should explain already here the GFP reporter system.

3. Page 20 line 61: The authors mention "RNA sequencing technology" but I suppose they meant RNA-sequencing analysis pipeline.

4. The authors should specific when they are talking about TE subfamilies or individual integrants in the text, legends and figures.

5. Fig. 4a. What is the value? If it is z-score, other type of normalized value as rpkm would make more sense.

6. Fig. 4c. What is the meaning of the triangle on the figure? The authors talk about the top 20 integrants but show actually top 20 subfamilies and plot the number of integrants.

7. Fig. 4d. Could the authors add to light gray some closely related family to show the specificity and remove the possibility of normalization bias due to the differential TE length between LINE and ERVs?

8. Fig. 5. The authors show all replicates, which is a good intention but makes it difficult to read. They should try to simplify and align better the legends of each track.

9. Fig. 5b. The authors should give statistics to support their claim that there is not much difference.

10. Page 23 line 31. The reference should go into the figure legend, and authors should indicate which mESC state the H3K4me3 data come from.

11. For convenience, could the authors make an xls file of their 500 supplementary table pages?

**Authors Response**

**Point-by-point responses to the reviewers’ comments:**

**Reviewer 1:**

This is impressive work, covering a wide range of techniques for discovering the molecular determinants of naive / primed states in murine cells. This topic has been covered by several previous posts, and therefore some parts are lacking in novelty, but many sections are new. Overall, this work highlights a critical point in initiated PSC, including that lower telomere maintenance results in telomere fragility, and that there is increased genomic instability related to retrotransposon activity. Overall, the scope of this work may make it a benchmark in the field of naive / primed pluripotency in rodents.

*Response: Thank you indeed for the encouraging and constructive comments for improving the quality of the manuscript.*

Major

1 / A major limitation of the study is the lack of indication of repeatability. For example, in Figure 3 E-G-H: how many times was the experiment repeated, how many experimental time points are shown in the figures? For bar charts, it is now common to overlay bar charts with bar charts. This should be preferred, as it explicitly states how many data points were used to build the graph. Similar note for Figure 6: The experiments in this figure are essential for the conclusion of the manuscript, however, there is evidence that each experiment was performed only once. Such a decisive experiment must be repeated at least three times.

*Response: These are important points. For most of the experiments, at least three times were repeated using two different cell lines. For telomere experiments in Figure E and G, we performed two biological repeats experiments using two cell lines. Now, number of repeats is added in the figure legend 3A, 3C, 3E, 3G, 3H and 3I. Based on your advice, we have re-drawn the related figures by the overlay bar charts using the original data. The new charts are shown in Figure 3A, B, C, G and H. We have performed additional experiments and further data analysis on WES like those of Figure 6. Three additional repeated experimental data from two cell lines at different background (OG2 and 129 lines from CBAxC57 and 129xC57background, respectively) are added in the revised Figure 6. The new results from seven cell line clones further consolidate our conclusion.*

2 / In FIG. 6E, the gains> = 5 are the most frequent events. It is understood that this is due to TE, but the authors should explain in more depth how this occurs. What is the percentage of CNV with at least one TE, and up to how much? Where available, do CNV boundaries provide any indication of the mechanism underlying the induction of CNV by TE? Discuss this part in more detail. Also, how do variations of CNV in primed cells compare to a more differentiated cell type? Discuss.

*Response: Thank you for the comments. In previous analysis for Figure 6, MEF cells served as reference samples and data for mutations or CNVs were obtained based on the comparison of naïve or primed ESC cells with MEF cells. We realize that this was not reasonable, because cultured MEF cells themselves could contain mutations and CNVs, and the outcome by comparison with MEFs could be misleading. Hence, in the new data analysis for mutations and CNVs, we used naïve ESCs as references and compare primed ESCs with naïve ESCs, because these naïve ESCs produced germline chimeras as well as TEC-pups that survived to adulthood, presumably indicating that the naïve ESCs are normal. Previously we had one cell line clone for the mutation analysis. We now have performed additional experiments on exomesequencing including six new cell line clones from two genetic background CBAxC57 and 129xC57. Methods for analysis are detailed in the revised method (page 15, line 10-22). In the new analysis, there is no arbitrarily defined CNVs the gains> = 5 (revised Figure 6).*

*We have adjusted the analytical pipeline for all seven samples. We use segmentation algorithm hmm and set threshold of 500bp and higher to define CNVs. To obtain the de novo CNVs from primed cells, we pooled all the naïve samples served as a reference, and filtered out the CNVs shared among four (CBAXC57) or three (129XC57) primed cell line clones. The percentage of CNV with at least one TE and different number of TEs now is shown in revised Figure 6D. The number of TEs in a CNV can be more than 100. We only find more CNVs and TEs in CNVs in primed cells, but cannot infer the underlying mechanisms based on our exome-sequencing data. Although a relatively large CNV contains one or many TEs, the CNV boundaries do not necessarily indicate the mechanism underlying the induction of CNV by TEs. (We discuss this point in Discussion (page 28, line 26-28). To address the last point, ESCs have higher DNA repair capacity compared to the somatic MEF cells (shown in Figure 1C). We speculate that the differentiated cells may be more prone to CNVs compared to the primed PSCs after long-term culture in vitro. But this certainly requires systematic experiments and sequencing data to directly compare differentiated cells with primed cells at the same passage stage.*

3 / In the abstract, the authors suggest that "telomere fragility and retrotransposon-induced genomic instability, associated with decreased DNA recombination repair, may be related to the compromised developmental potency of primed PSCs, noticeably distinguishable from naive PSCs ". This should be discussed in the discussion section. It should be noted that genetic instability is only one of the many hypotheses that could explain the low developmental power of initiated PSCs. Other hypotheses include the differential cell cycle with ICM, poor survival in the ICM setting, or decreased differentiation properties.

*Response: Thank you for the advice. We have discussed these points including genetic instability, cell cycle, apoptosis, and differentiation properties on developmental potency of PSCs in Discussion (Page 29, line 1-4; page 2 summary, line 16-20). We have compared our RNA-seq data of naïve and primed cells with the in vivo ICM and post-implantation epiblast counterpart (data from [1]). Indeed, naïve cells are clustered with ICM and primed cells clustered with the epiblast (revised Figure S3E,F). The cell cycle and cell survival program in naïve cells resemble ICM, while the primed state cells look more like post-implantation epiblast. Moreover, primed state cells express marker genes, like T, Gata6 and Sox17 (Figure S1A), and other genes such as Eomes and Gsc (data shown below) for mesendoderm differentiation [2]. Hence, the developmental difference between naïve and primed cells can be explained by the comprehensive factors, including the genomic stability, differential cell cycle, cell survival program and differential differentiation properties (in Discussion page 28-29).*

**Reviewer 2:**

In "Elevated retrotransposon activity and genomic instability in primed pluripotent stem cells", Fu et all aim at elucidating mechanisms that could be involved in the lower developmental potency of primed pluripotent stem cells, when compared to naïve embryonic stem cells.

While the subject is an important one, I feel that this manuscript is too speculative and lacks functional evidence for the authors conclusions (which are many, in their Discussion Section). In addition, by combining telomere data with retrotransposon analysis, with the lack of any mechanistical reasoning to do so, the manuscript becomes unnecessarily long and descriptive. A few more specific points bellow:

*Response: Thank you for your constructive comments which are very helpful for improving the manuscript. Your specific points are heeded, and additional experiments have been performed to address the concerns.*

1)	Figure 1 doesn't add anything novel to the field. It is simply a description of gene expression profiles in naïve vs primed, which has been described many times before.

*Response: These are great points. These data were presented to make sure that our primed and naïve ESCs are consistent with previous reports on characterization of primed and naïve PSCs at cellular and molecular levels, such that these reliable, distinct cell types can be reliably used for subsequent studies. Based on your advice, we now focus on the new data about elevated recombination and DNA repair pathways revealed in naïve ESCs, compared to primed ESCs, and merge part of the RNA-seq data with Supplementary Figure 1. Based on the molecular changes, we hypothesized that naïve cells have higher capacity in DNA repair than do primed cells. Hence, we have performed additional experiments to functionally demonstrate the significance of the pathways in naïve and primed cells. The cells were induced to DNA damage by exposure to H2O2 or etoposide, and then analyzed DNA damage responses by commonly used markers 53BP1 and γH2AX [3]. DNA damage foci increased immediately in naïve and primed ESCs after exposure to 2.5 μM etoposide or 5 mM H2O2 for 2 h. Notably, 20 h following exposure to H2O2 or etoposide, DNA damage foci indicated by either 53BP1 or γH2AX were decreased in naïve ESCs. However, the recovery from DNA damage response appeared to be much slower in primed ESCs, which displayed much more DNA damage foci than did naïve ESCs (data added in revised Figure 1E-H and Figure S1J). These data support the notion that the naïve cells have stronger DNA repair capacity than do primed state cells. The experiments are repeated using two different ESC lines and the results are consistent (revised text, page 18, line 4-16, amended method, page 7, line 17-22).*

2)	The authors argue (from GO analysis) that recombination and DNA damage pathways are reduced in the primed state, when compared to the naïve state. The authors reach this conclusion because RNASeq analysis show lower expression of HR, MMR and NER genes. However, the expression levels of these genes alone is far from being sufficient to reach such a conclusion. The authors should challenge both cell types to IR, ROS and UV light and quantify rates of DNA repair and cell survival.

*Response: Thank you for the advice. We agree that the experiments you suggested are absolutely important to strength the conclusion about reduced DNA repair in primed state. Hence, we have performed experiments on DNA damage responses by challenging the naïve and primed cells to DNA damage reagents including H2O2 induced ROS and etoposide, also commonly used for damaging DNA, which we previously had some experience, although IR is not available in our lab, and we did not have experience on using UV light. These results demonstrate that DNA repair capacity is reduced in primed state compared to naïve state, and the data are provided in revised Figure 1E-H and S1J, and related text (page 18, line 4-16). Methods also are added on page 7, line 17-22.*

3)	Authors argue a possible decrease in the OXPHOS/Glycolytic pathway in the transition from naïve to primed based on RNAseq. At the very minimal a Seahorse analysis should be performed to validate this claim.

*Response: Based on your advice, we have performed functional experiments to look at OXPHOS pathway in naïve and primed state. It appears that OXPHOS pathway is reduced in the transition from naïve to primed state. The new data is provided in revised Figure 2G, and the revised text on page 19, line 3-6 (Methods are added on page 11, line 27-33).*

4)	Expression of TERT and TERC are similar (Figure 3), and telomerase activity assays (unfortunately performed by PCR) reflect that. On the same figure, authors claim telomere elongation in naïve and not in primed cells. The difference however seems to be minimal and is difficult to analyze as we only see relative data (Figure 3C) and not telomere length by itself. The authors should show telomere length assays by TRF or STELLA. Arguably the Q-FISH analysis (3D/E) serves this purpose, but the elongation rates between that and 3C are discordant, and the standard deviation pretty significant.

*Response: We also detected the telomerase activity by ELISA assay (Figure 3B) in addition to TRAP assay (Supplementary Figure 5C). Given that the telomere length alterations are important findings of this study, we measured telomere lengths using three commonly used methods available, real-time qPCR method (Figure 3C), quantitative telomere FISH (Figure 3D, E and Supplementary Figure 5D, E) and TRF by southern blot (Supplementary Figure 5F). The standard deviation of telomere length by Q-FISH analysis is usually pretty large which can indicate the telomere length in single cell at mitosis and the telomere length heterogeneity in a large cell population, and this phenomenon is shown in the original paper [4] and other papers as for an example [5, 6]. Together, these data consistently demonstrated that telomeres elongate in naïve cells, whereas telomere length only can be maintained in the primed cells at early passages, and gradually shorten with increasing passages.*

5)	On page 19, line 25, the authors write: "Regardless of the mechanisms, this, along with the declined DNA recombination repair capacity […]". The fact is that one cannot disregard mechanism when making claims like this. Also, the authors don't show declined DNA repair capacity in any of their figures. Only expression data. Those are not the same. Too speculative.

*Response: We appreciate your comments. Now based on the new experimental data shown above (revised Figure 1E-H, Figure S1J), the primed state exhibits declined DNA repair capacity, unlike the naïve state. Hence, accordingly, the text is revised on page 20, line 1-4.*

6)	Page 20, line 50: The authors claim that "Reduced telomere recombination activity corroborates robust telomere lengthening in naïve PSCs". Again, functional data is lacking to support this. Manuscript has many associations, but a lack functional assays to support most of their claims.

*Response: Telomere chromosome orientation FISH (CO-FISH) allows the assessment of the relative efficiency of lagging versus leading strand telomere replication and thus provides information of genetic recombination that often occurs near the telomere, namely sister chromatid exchange (T-SCE)[7, 8]. We have conducted CO-FISH experiments using three different cell lines shown in Figure 3I and S5H, indicating that the telomere recombination activity shown by T-SCE is notably higher in naïve cells than in primed cells. T-SCE is absent or minimal in primed cells. Higher T-SCE suggests higher telomere recombination activity. We have added two references to interpret the T-SCE as indicative of telomere recombination (revised text, page 20, line 11-13). Unfortunately, we had a writing mistake for the sentence, there should be “Increased telomere recombination activity corroborates robust telomere lengthening in naïve PSCs “, corrected in this revision (revised text, page 21, line 13-14). We apologize for this error.*

7)	Page 23, line 36: Authors claim "These data suggested that diverse epigenetic modifications orchestrate in reducing naïve pluripotency gene expression and suppressing specific 2C genes in primed cells." This is too vague; no validation, just speculation.

*Response: Here in this revision, we further analyze the effects of knockout of Setdb1, G9a and Dnmt3b as H3K9me3, H3K9me1/2 and DNA methyltransferases, respectively, based on the database published previously by other laboratories [9-11]. The naïve genes and 2C genes including Dux were upregulated by Setdb1 KO or G9a KO in naïve mESCs (new Figures S8 G-J). Fewer 2C genes were upregulated by Dnmt3b KO in naïve mESCs, while naïve state genes were upregulated by Dnmt3b KO (new Figures S8 K and L). These results are consistent with the data shown in Figure 5B, suggesting that 2C genes are mainly regulated by H3K9 methylation in naïve and primes PSCs and less likely by DNA methylation, which is consistent with the previous paper [1]. The new figures and results are added in the Figure S8 G-L. These data suggested that H3K9me3 and DNA methylation modifications regulate naïve pluripotency gene and 2C gene expression in naïve mESCs (revised text, page 23, line 20-28).*

8)	An important conclusion of the authors is that high Line1 and Alu activity, together with reduced telomere length in primed cells reduce the ability of primed ESCs to contribute to chimera. Again, no functional data to directly test this hypothesis is presented. If the telomeres get shorter and there is increased retrotransposition of Alu/L1 in primed cells with serial passage, then the chimera-forming ability of these cells at early passages should be similar to naïve cells, and decrease only after serial passaging. Authors should compare the chimera-forming ability of early (P5) versus late (P>30) primed ES cells and confirm this claim.

*Response: These are quite reasonable points. We injected early passage (P12+4) primed mEpiSCs for chimera assay, and none of them can generate chimeras (Figure S2A and figure legend). It is likely that the chimera formation ability expectedly will be also low for late passage (P>30) primed ESCs. Now, based on our pervious data and additional experimental data and analysis in this revision (e.g. Figure S1H, Figure 2G, Figure S3E-F), now it seems to be reasonable to conclude that complex factors including cell cycle dysfunction, mitochondria defects, increased apoptosis, differentiation properties, together with high retrotransposon activities and telomere damage can reduced the ability of primed ESCs to contribute to chimera, and thus lower developmental potential (revised text, page 28-29).*

**Reviewer 3:**

This work is a remarkable resource, consolidated by deeply analyses and many functional experiments, especially regarding telomeres. If the authors had added peri-centromic to telomeric regions and transposons, they would have truly performed a thorough analysis of DNA repeats, which would have given to their study a more definitive character ("mouse genomic repeat behaviors during naive to primed embryonic stem cells transition.") The part on TEs is not fundamentally novel, and some statements should be toned down, especially some of their conclusion about genomic stability.

*Response: Thank you for the encouraging comments. Your advice is very important for improving the manuscript. Some improper statements are revised and toned down as to summary about genomic instability.*

*We have analyzed the peri-centromic to telomeric regions and transposons behaviors by our RNA-seq data. However, as shown below, we could not find significant pattern in terms of TE position within peri-centromic to telomeric regions between naïve and primed mESCs. So, in the revision we do not extend more on this analysis.*

*legend: The enrichment significance of differentiated expressed TEs in nearby region of telomere/centromere (tel/centr) and telomere-only (tel-only) according to the annotation from UCSC. P value was calculated by fisher’s exact test. -log10 (p value) more than 1.3 (-log10(p value = 0.05)) is considered to be significant.*

Major comments:

1. Fig. 3. In addition to loss-of-function experiments, is gain of function sufficient to change the telomere's phenotype?

*Response: We agree on your point. We showed that loss of Dmc1 leads to notable telomere shortening. Rad51 KO has only minimal effects on telomeres. Thus, based on your advice, for the revision we have attempted to overexpress Dmc1 in the Dmc1 KO cell lines and see if the telomere defects can be rescued, but failed in achieving the Dmc1 overexpressed cells for further studies, even though we performed this experiment for several times. This is very surprising though. Due to the revision deadline approaching even after we requested to extend the deadline, we feel that it is a pity to have no relevant data to be included in this revised manuscript. However, we speculate that perhaps Dmc1 is very important for telomere length regulation, and too much Dmc1 may also be detrimental to cell survival, so we plan to do more extensive studies on potential role of Dmc1 in telomere regulation and to understand its mechanisms. This could be a complete story for another paper.*

2. Fig. S7b. How do the authors reconcile their observations and previous reports indicating that naive mESC display decreased levels of DNA methylation and H3K27me3? This should be strongly discussed.

*Response: We actually did not show decreased levels of DNA methylation and H3K27me3 in naive mESC (Figure S7). Original results: “By immunofluorescence microscopy and dot blot assay, 5mC level did not change, but 5hmC level was decreased in primed cells (Figures S7B-S7D).” (page 21, lines 9-11). “Initially we examined the expression pattern of potential histone epigenetic modifications, including H3K9me3, H3K27me3, and H3K27ac by immunofluorescence microscopy, which revealed no significant difference between naïve and primed cells (Figure S7E). H3K9me3 exhibited typical foci consistent with DAPI-stained heterochromatin, and H3K27ac and H3K27me3 showed similar nuclear distribution in naïve and primed cells.” (page 2, line 30, lines 1-6 on page 23).*

3. Fig. 5 and page 23 line 33. The authors did not need to "infer" that Dux4 might regulate 2-cell stage genes: that had already been demonstrated and published.

*Response: This sentence is now deleted.*

4. Fig. S9. The authors re-analyzed papers published years ago, when sequencing technology was still sub-optimal for proper mapping of retro-transposons. Moreover, the authors should also add up all reads from ChIP-seq and RNA-seq (including multimap) over each subfamily to make sure they do not just lose them when mapping uniquely. The authors may want also to re-analyze GSE95720, which represents a more recent dataset of KAP1 KD obtained using longer sequencing reads.

*Response: Thank you for advice. Accordingly, we have re-analyzed the GSE95720 database and added the new analysis data and replaced the old data in the revised Figure S9 B, C and text page 25, lines 18-26. The new database better supports our previous analysis. We used hisat2 to align reads directly to the TE consensus sequences (from Dfam database) and calculate the differentially expressed TEs by our RNA-seq data and the new KAP1 KD database of GSE95720 as below. In addition, we used bowtie2 to align reads of ChIP-seq data on IAPEz-int and L1Md_T consensus sequences as below. The results are consistent with our previous analysis, further supporting the reliability of the method for analysis of TEs.*

5. Fig. 6. How do the authors discriminate events which might appear randomly in individual cells from a bulk population? Isolation and sequencing of multiple clones should be performed. Are the observed retro-transposable elements enrichment difference between naive and primed ESC significant? Are there de novo insertions specific to primed versus naïve ESC? We are really not sure that these analysis support the claim of the authors.

*Response: We fully understand the concerns and it is important to address the issue. Previously we had one cell line clone for the mutation analysis. We now have performed additional experiments on exome-sequencing including six new cell line clones from two genetic background CBAxC57 and 129xC57. Methods for analysis are detailed in the revised method (page 15, line 10-22). In previous analysis for Figure 6, MEF cells served as reference samples and data for mutations or CNVs were obtained based on the comparison of naïve or primed ESC cells with MEF cells. We realize that this was not reasonable, because cultured MEF cells themselves could contain mutations and CNVs, and the outcome by comparison with MEFs could be misleading. Hence, in the new data analysis for mutations and CNVs, we used naïve ESCs served as references and compare primed ESCs with naïve ESCs, because these naïve ESCs produced germline chimeras as well as TECpups that survived to adulthood, presumably indicating that the naïve ESCs are normal. We have adjusted the analytical pipeline for all seven samples. We use segmentation algorithm hmm and set threshold of 500bp and higher to define CNVs. To obtain the de novo CNVs from primed cells, we pooled all the naïve samples served as a reference, and filtered out the CNVs shared among four (CBAXC57) or three (129XC57) primed cell line clones. The percentage of CNV with at least one TE and different number of TEs now is shown in revised Figure 6D. The number of TEs in a CNV can be more than 100. We only find more CNVs and TEs in CNVs in primed cells, but cannot infer the underlying mechanisms based on our exome-sequencing data. Although a relatively large CNV contains one or many TEs, the CNV boundaries do not necessarily indicate the mechanism underlying the induction of CNV by TEs. (We discuss this point in Discussion (page 28, line 26-28).*

Minor comments:

1. Some figures would deserve higher resolution.

*The original figures in TIFF or JPG were in high resolution. The resolution is reduced somehow during PDF conversion. We now save jpg figure file directly as PDF for review purpose.*

2. Page 16 line 6: The authors should explain already here the GFP reporter system.

*We have explained the Oct4-driven GFP reporter on Page 16 line 5-7 (revised text, page 5, lines 7-8).*

3. Page 20 line 61: The authors mention "RNA sequencing technology" but I suppose they meant RNA-sequencing analysis pipeline.

*Thank you for pointing out this. We have revised this sentence*

4. The authors should specific when they are talking about TE subfamilies or individual integrants in the text, legends and figures.

*These are now clarified in the text highlighted in yellow, legends and figures (Figure 4C, and legend 4C).*

5. Fig. 4a. What is the value? If it is z-score, other type of normalized value as rpkm would make more sense.

*The value is z-score. We have replotted the heatmap using log10(FPKM). (revised Figure 4A).*

6. Fig. 4c. What is the meaning of the triangle on the figure? The authors talk about the top 20 integrants but show actually top 20 subfamilies and plot the number of integrants.

*We used the triangles to highlight the high expression of MERVL in naïve mESCs and this is confirmed in Figure 4E. We have added the interpretation of triangle in the figure legend. We have also corrected the statement in Figure 4C legend.*

7. Fig. 4d. Could the authors add to light gray some closely related family to show the specificity and remove the possibility of normalization bias due to the differential TE length between LINE and ERVs?

*As it is unclear and complicated to plot many related families in one scatter blot graph simultaneously, we have replaced the scatter plot with the boxplot of TE loci for several sequence-similar TE families. Now the figure appears clearer. The log10 (FPKM+0.01) is used for relative expression levels (revised Figure 4D and figure legend).*

8. Fig. 5. The authors show all replicates, which is a good intention but makes it difficult to read. They should try to simplify and align better the legends of each track.

*Thank you for pointing out this. We have simplified and aligned better the legends of each track. (revised Figure 5 and S8).*

9. Fig. 5b. The authors should give statistics to support their claim that there is not much difference.

*We used Wilcoxon signed-rank Test to calculate the significance of difference. Statistics are now added in Figure 5B.*

10. Page 23 line 31. The reference should go into the figure legend, and authors should indicate which mESC state the H3K4me3 data come from.

*We now put the reference in the figure legend and indicate source data of H3K4me3 for the naïve mESCs (serum/Lif). (Page 43, line 19-21)*

11. For convenience, could the authors make an xls file of their 500 supplementary table pages?

*The problem could be caused by processing of uploading files. We now re-submit the xls file including differentially expressed genes and TEs.*

*References: 1.Wang C, Liu X, Gao Y, Yang L, Li C, Liu W, Chen C, Kou X, Zhao Y, Chen J, et al: Reprogramming of H3K9me3-dependent heterochromatin during mammalian embryo development. Nat Cell Biol 2018, 20:620-631.*

*2.Alexanian M, Maric D, Jenkinson SP, Mina M, Friedman CE, Ting CC, Micheletti R, Plaisance I, Nemir M, Maison D, et al: A transcribed enhancer dictates mesendoderm specification in pluripotency. Nat Commun 2017, 8:1806.*

*3.Zimmermann M, de Lange T: 53BP1: pro choice in DNA repair. Trends Cell Biol 2014, 24:108-117.*

*4.Poon SS, Martens UM, Ward RK, Lansdorp PM: Telomere length measurements using digital fluorescence microscopy. Cytometry 1999, 36:267-278.*

*5.Marion RM, Strati K, Li H, Tejera A, Schoeftner S, Ortega S, Serrano M, Blasco MA: Telomeres acquire embryonic stem cell characteristics in induced pluripotent stem cells. Cell Stem Cell 2009, 4:141-154.*

*6.Gonzalo S, Jaco I, Fraga MF, Chen T, Li E, Esteller M, Blasco MA: DNA methyltransferases control telomere length and telomere recombination in mammalian cells. Nat Cell Biol 2006, 8:416-424.*

*7.Ourliac-Garnier I, Londono-Vallejo A: Telomere Strand-Specific Length Analysis by Fluorescent In Situ Hybridization (Q-CO-FISH). Methods Mol Biol 2017, 1587:41-54.*

*8.McKenna MJ, Robinson E, Goodwin EH, Cornforth MN, Bailey SM: Telomeres and NextGen CO-FISH: Directional Genomic Hybridization (Telo-dGH). Methods Mol Biol 2017, 1587:103-112.*

*9.Neri F, Rapelli S, Krepelova A, Incarnato D, Parlato C, Basile G, Maldotti M, Anselmi F, Oliviero S: Intragenic DNA methylation prevents spurious transcription initiation. Nature 2017, 543:72-77.*

*10.Mozzetta C, Pontis J, Fritsch L, Robin P, Portoso M, Proux C, Margueron R, Ait-SiAli S: The histone H3 lysine 9 methyltransferases G9a and GLP regulate polycomb repressive complex 2-mediated gene silencing. Mol Cell 2014, 53:277-289.*

*11.Wu K, Liu H, Wang Y, He J, Xu S, Chen Y, Kuang J, Liu J, Guo L, Li D, et al: SETDB1-Mediated Cell Fate Transition between 2C-Like and Pluripotent States. Cell Rep 2020, 30:25-36 e26.*

**Second round of review**

**Reviewer 1**

The authors answered satisfactorily the issues I raised. The new experiments carried out in response to all the reviewers’ remarks added value to the manuscript, which is now improved.

**Reviewer 2**

The authors have adequately addressed many of my concerns. Specifically, the addition of functional data to corroborate many of the previous claims make this manuscript a lot stronger and significantly broadens its scope. During the period that this paper was in review/under revision, different manuscripts were published that also addressed how telomere maintenance is distinct between cells in different states. I believe these manuscripts should be incorporate in the Discussion section, as they go in line with the claims of this paper. They are:

Telomere erosion in human pluripotent stem cells leads to ATR-mediated mitotic catastrophe (Vessoni et al, JCB, 2021)

TRF2-mediated telomere protection is dispensable in pluripotent stem cells (Marta Markiewicz-Potoczny et al, Nature, 2021)

TRF2-independent chromosome end protection during pluripotency (Ruiz et al, Nature, 2021)

**Reviewer 3**

While the authors answered some of our previous concerns, questions remain:

1.Fig. 3. In addition to loss-of-function experiments, is a gain of function sufficient to change the telomere's phenotype? A rescue experiment still seems to be the proper experiment to do.

2.Fig. S7b. How do the authors reconcile their observations and previous reports indicating that naive mESC displays decreased DNA methylation levels and H3K27me3? This should be intensely discussed.

3.Fig. S9. The authors re-analyzed papers published years ago, when sequencing technology was still sub-optimal for proper mapping of retro-transposons. Moreover, the authors should also add up all reads from ChIP-seq and RNA-seq (including multimap) over each subfamily to make sure they do not just lose them when mapping uniquely. The authors may want also to re-analyze GSE95720, which represents a more recent dataset of KAP1 KD obtained using longer sequencing reads.

The authors are still analyzing the data without using unique read mapping (an option that is not the default parameters of bowtie2 and HiSat). This should be performed before reaching any conclusion.

4.Fig. 6. How do the authors discriminate events which might appear randomly in individual cells from a bulk population? Isolation and sequencing of multiple clones should be performed. Are the observed retrotransposable elements enrichment difference between naive and primed ESC significant? Are there de novo insertions specific to primed versus naïve ESC?

**Authors Response**

**Point-by-point responses to the reviewers’ comments:**

**Reviewer 2:**

The authors have adequately addressed many of my concerns. Specifically, the addition of functional data to corroborate many of the previous claims make this manuscript a lot stronger and significantly broadens its scope. During the period that this paper was in review/under revision, different manuscripts were published that also addressed how telomere maintenance is distinct between cells in different states. I believe these manuscripts should be incorporate in the Discussion section, as they go in line with the claims of this paper. They are:

Telomere erosion in human pluripotent stem cells leads to ATR-mediated mitotic catastrophe (Vessoni et al, JCB, 2021)

TRF2-mediated telomere protection is dispensable in pluripotent stem cells (Marta Markiewicz-Potoczny et al, Nature, 2021)

TRF2-independent chromosome end protection during pluripotency (Ruiz et al, Nature, 2021)

*Response: Thank you for the constructive comments and advice on discussion of the relevant recent papers. Now we have discussed the findings of these three references in the Discussion (Page 17).*

*“In consistency, telomere erosion in human pluripotent stem cells conventionally known as primed state of pluripotency induces DNA damage response and leads to ATRmediated mitotic catastrophe [82]. Also, mESCs depleted for telomere maintenance protein TRF2 elicit minimal or only mild DNA damage response, in contrast to nonpluripotent or differentiated cells showing that TRF2-mediated telomere protection is dispensable in pluripotent stem cells [83, 84]. Notably, TRF2 depletion in mESCs activates a totipotent-like two-cell-stage transcriptional profile including high levels of Zscan4 and the upregulation of Zscan4 reduces DNA damage and elongates telomeres in the absence of TRF2 [84], providing additional evidence in supporting importance of Zscan4 in pluripotent stem cells.”*

**Reviewer 3**

While the authors answered some of our previous concerns, questions remain:

1.Fig. 3. In addition to loss-of-function experiments, is a gain of function sufficient to change the telomere's phenotype? A rescue experiment still seems to be the proper experiment to do.

*Response: In the Fig. 3 Distinct Telomere Maintenance in Naïve and Primed Cells, we compared the differences in telomere length, telomere recombination and fragile telomeres between naive and primed ESCs. Naive ESCs exhibited longer telomere and higher telomere recombination, and fewer fragile telomeres, compared with those of primed ESCs. Actually, no loss-of-function experiments were performed in the Fig. 3 data. So, we do not have in mind specific gain of function or rescue experiments to change the telomere's phenotypes. These phenotypes can be caused by many factors which could take longer time to define, but we agree that these definitely will be good scientific questions that require systematic experiments to address in the future research.*

*In the first round of review, Your major concern was: “1. Fig. 3. In addition to loss-offunction experiments, is gain of function sufficient to change the telomere's phenotype?”*

*Our response: We agree on your point. We showed that loss of Dmc1 leads to notable telomere shortening. Rad51 KO has only minimal effects on telomeres. Thus, based on your advice, for the revision we have attempted to overexpress Dmc1 in the Dmc1 KO cell lines and see if the telomere defects can be rescued, but failed in achieving the Dmc1 overexpressed cells for further studies, even though we performed this experiment for several times. This is very surprising though. Due to the revision deadline approaching even after we requested to extend the deadline, we feel that it is a pity to have no relevant data to be included in this revised manuscript. However, we speculate that perhaps Dmc1 is very important for telomere length regulation, and too much Dmc1 may also be detrimental to cell survival, so we plan to do more extensive studies on potential role of Dmc1 in telomere regulation and to understand its mechanisms. This could be a complete story for another paper.”*

*Now by looking at the figures, we think that your comment might be about the data presented in Figure S6. You may want to know whether exogenously expressing Dmc1 was just toxic in WT cells, or whether we tried to complement the KO cells and also failed. The latter, called rescue experiment, would have helped ascertain that the phenotype was not an off target effect. As it cannot be addressed experimentally (see previous response above), we now discuss this shortcoming in the Discussion (Page 17).*

*“Increasing evidence established that Zscan4 is critical to telomere elongation and maintenance of naïve mouse ESCs. However, mechanisms underlying Zscan4 mediated telomere elongation remain elusive. Telomeres shortened after Dmc1 KO in naïve ESCs and slightly shortened after Rad51 KO (Additional file 1: Figure S6e, f), implying that DNA recombination is involved in telomere maintenance. We have attempted to exogenously overexpress Dmc1 in the Dmc1 KO naïve cell lines but failed in achieving the Dmc1 rescue cells. As we did not overexpress Dmc1 in WT naïve cells, we cannot conclude whether overexpression of Dmc1 is toxic to the cells or whether this phenotype is an off-target effect. Also, it is likely that the telomere phenotype in primed state cells may be caused by comprehensive factors. Overexpression of a single factor alone may not be sufficient to rescue telomere phenotype in primed state cells. It will be necessary to systematically investigate telomere function and regulation in naïve and primed PSCs in future experiments.”*

2.Fig. S7b. How do the authors reconcile their observations and previous reports indicating that naive mESC displays decreased DNA methylation levels and H3K27me3? This should be intensely discussed.

*Response: The differences between naïve mESCs in this manuscript and other papers in terms of DNA methylation and H3K27me3 could be due to the differences in culture conditions. serum+LIF condition was used in our study reported here and 2iL medium in previous reports.*

*We intensively introduced the culture condition effects in the Background, ‘Moreover, a ‘ground state’ beyond naïve state has been achieved by culture under 2i/LIF condition (2i, GSK3, and MEK1/2 inhibitors) [19]. 2i reduces the DNA methyltransferases Dnmt3a/3b to promote hypomethylation and enhances Tet1/2 activity (consequently, increased 5-hydroxymethylcytosine (5hmC) levels) and ‘passive’ loss of DNA methylation, resulting in a distinct transcriptional and epigenetic state that includes uniform expression of key pluripotency factors, such as Nanog and Prdm14 [20, 21]. Ground state cells exhibit lower expression of lineage affiliated genes, reduced prevalence at promoters of the repressive histone modification H3K27me3, and fewer bivalent domains, which are thought to mark genes poised for either up- or downregulation [22]. Furthermore, female PSCs under the ground state or naïve state show both X chromosome activation, but inactivate one of the X chromosomes under primed state [18]. mESCs under 2i culture conditions also can produce all-ESC pups but prolonged inhibition with 2i could damage the developmental potential [23-25].’ (Page 3). We now further clarify our naïve ESC culture conditions in the first paragraph in Discussion (Page 16).*

3.Fig. S9. The authors re-analyzed papers published years ago, when sequencing technology was still sub-optimal for proper mapping of retro-transposons. Moreover, the authors should also add up all reads from ChIP-seq and RNA-seq (including multimap) over each subfamily to make sure they do not just lose them when mapping uniquely. The authors may want also to re-analyze GSE95720, which represents a more recent dataset of KAP1 KD obtained using longer sequencing reads.

The authors are still analyzing the data without using unique read mapping (an option that is not the default parameters of bowtie2 and HiSat). This should be performed before reaching any conclusion.

*Response: According to your suggestion, we re-analyzed the Kap1 KD RNA-seq data in GSE95720 (New Figure S9). We added up all reads from RNA-seq (including multimap) over each subfamily. It turns out that IAP subfamilies including IAPEz-int and IAPA_MM-int and L1 subfamily L1Md_A are significantly upregulated after Kap1 KD (Figure S9C). We also added up all reads from ChIP-seq (including multimap) over each subfamily in related study from GSE94323. Likewise, the data shows that IAP subfamilies including IAPEz-int and IAPA_MM-int and L1 subfamilies including L1Md_T and L1Md_A are repressed by Kap1-mediated H3K9me3 (Figures S9D-G). Additionally, we compared the same database by both uniquely mapped reads and multi-mapped reads. The results from two methods are consistent (Figures S9C, D, G). Based on the new analysis, we have revised the text (Page 14-15) and Method (Page 28, 29).*

4.Fig. 6. How do the authors discriminate events which might appear randomly in individual cells from a bulk population? Isolation and sequencing of multiple clones should be performed. Are the observed retrotransposable elements enrichment difference between naive and primed ESC significant? Are there de novo insertions specific to primed versus naïve ESC?

*Response: Based on your advice in the first-round revision, we performed additional experiments on exome-sequencing using six new cell line clones from two genetic background CBAxC57 and 129xC57. Methods for analysis were detailed in the revised method (Page 30). Thus, multiple clones were performed. However, these data cannot discriminate events which might appear randomly in individual cells from a bulk population.*

*In the mutation analysis by exome-seq, we applied the naïve ESCs served as references and compared the primed with naïve ESCs, because the naïve ESCs were able to produce germline chimeras as well as TEC-pups, indicating that the naïve ESCs are genomic stable. By such comparison with naïve ESCs, any insertions and the TEs contained within CNVs in primed ESCs presumably are considered as de novo and specific (Figures 6B-I).*

*Our new results showed that six out of seven primed clones from two background have more CNVs compared to the naïve clones, and these CNVs contain similar families of TEs like L1, Alu, B2, B4, etc. The variable number of CNVs and TEs within CNVs indicates the heterogeneity of different clones (Table S5 and Page 15). Due to big variations in CNVs (less than 10 to nearly 100, Figure 6B,C) and the TEs within CNVs (few as 3 to nearly 600 gains and about 200 to more than 4000 loss, Figure 6E) among the clones in primed ESCs compared to naïve ESCs served as normal control, the data for each independent clone is not appropriate* *for productive statistical analysis.*

**Third round of review**

**Reviewer 3:**

Thanks for addressing my last comments
